# A comprehensive prognostic and immunological analysis of ephrin family genes in hepatocellular carcinoma

**DOI:** 10.3389/fmolb.2022.943384

**Published:** 2022-08-16

**Authors:** Shenglan Huang, Cairong Dong, Jian Zhang, Shumin Fu, Yaqin Lv, Jianbing Wu

**Affiliations:** ^1^ Department of Oncology, The Second Affiliated Hospital of Nanchang University, Nanchang, Jiangxi, China; ^2^ Jiangxi Key Laboratory of Clinical and Translational Cancer Research, Nanchang, Jiangxi, China; ^3^ Department of Hepatobiliary Surgery, The Second Affiliated Hospital of Nanchang University, Nanchang, Jiangxi, China

**Keywords:** ephrin family, prognosis, immune, biomarker, hepatocellular carcinoma

## Abstract

**Background:** Ephrins, a series of Eph-associated receptor tyrosine kinase ligands, play an important role in the tumorigenesis and progression of various cancers. However, their contributions to hepatocellular carcinoma (HCC) remain unclear. Thus, we aimed to explore their prognostic value and immune implications in HCC.

**Methods:** Multiple public databases, such as TCGA, GTEx, and UCSC XENA, were used to analyze the expression of ephrin genes across cancers. Kaplan-Meier analysis and Cox regression were used to explore the prognostic role of ephrin genes in HCC. A logistic regression model was utilized to evaluate the association between ephrin gene expression and clinical characteristics. Gene set enrichment analysis (GSEA) was conducted to elucidate their potential biological mechanisms. Various immune algorithms were utilized to investigate the correlation between ephrin genes and tumor immunity. We also analyzed their association with drug sensitivity, and gene mutations. Finally, RT–qPCR was performed to validate the expression of ephrin family genes in HCC cells and clinical tissues.

**Results:** The expression of EFNA1, EFNA2, EFNA3, EFNA4, EFNB1, and EFNB2 was upregulated in most cancer types, while EFNA5 and EFNB3 was downregulated in most cancers. In HCC, the expression levels of EFNA1, EFNA3, EFNA4, EFNB1, and EFNB2 were significantly higher in tumor tissues than in normal tissues. High expression of EFNA3, EFNA4, and EFNB1 was associated with tumor progression and worse prognosis in HCC patients. The expression of EFNA3 and EFNA4 was negatively associated with the stromal/ESTIMATE scores, while EFNB1 was positively correlated with the immune/stromal/ESTIMATE scores. Moreover, these ephrin genes were closely relevant to the infiltration of immune cells, such as B cells, CD4^+^ T cells, CD8^+^ T cells, neutrophil cells, macrophage cells, and dendritic cells. EFNB1 expression was positively associated with most immune-related genes, while EFNA3/EFNA4 was positively related to TMB and MSI. In addition, EFNA3, EFNA4, and EFNB1 were related to drug sensitivity and affected the mutation frequency of some genes in HCC.

**Conclusion:** EFNA3, EFNA4, and EFNB1 are independent prognostic factors for HCC patients and are closely correlated with tumor immunity, which may provide a new direction for exploring novel therapeutic targets and biomarkers for immunotherapy.

## Introduction

Hepatocellular carcinoma (HCC) is one of the most prevalent malignant tumors and ranks fourth among the most common causes of cancer-related death worldwide (Yang J. D. et al., 2019). Several factors, including chronic hepatitis B and hepatitis C, cirrhosis, alcohol abuse, nonalcoholic fatty liver disease and exposure to dietary toxins such as aristolochic acid and aflatoxins, remarkably increase the occurrence risk of HCC (Yang J. D. et al., 2019; Kulik and El-Serag, 2019; Llovet et al., 2021). Early-stage HCC can be treated curatively by surgical excision, local ablation, or liver transplantation. However, the majority of HCC patients are diagnosed at an advanced stage and are unsuitable for curative treatments (Fitzmaurice et al., 2017). Multiple kinase inhibitors and immune checkpoint inhibitors (ICIs) have been proven to be effective treatment options for advanced-stage HCC in recent years. The prognosis of HCC remains unsatisfactory, with cancer-specific mortality still increasing in many countries and an overall 5-year survival rate of only approximately 18% (Galle et al., 2021). The poor prognosis and high mortality of HCC patients are mainly attributed to molecular heterogeneity and the lack of early and effective indictive markers (Yang J. D. et al., 2019; Kulik and El-Serag, 2019). Thus, exploring reliable prognostic biomarkers and effective therapeutic targets is critically important to improve the clinical outcomes of HCC patients.

Erythropoietin-producing hepatocellular carcinoma (Eph) and Eph receptor interacting ligands (ephrins, EFNs) are the largest family of membrane-bound receptor tyrosine kinases, which consist of fourteen Eph receptors and eight ephrin ligands (Kullander and Klein, 2002). Ephs and ephrins are widely expressed on the surface of various cells. Characteristic bidirectional signaling is induced through Eph–ephrin interactions in receptor- and ligand-expressing cells; Eph receptors activated by ephrin ligands are referred to as “forward signaling,” resulting in phosphorylation of the receptors and activation of downstream signaling molecules, while “reverse signaling” is defined as Eph receptor-mediated activation of ephrin ligands (Héroult et al., 2006; Lin et al., 2021). The Eph-ephrin complexes are involved in a wide spectrum of physiological and pathological processes and affect cell biological functions during development, such as neurogenesis and angiogenesis, cell proliferation and differentiation, cell segregation, cellular motility and adhesion (Brückner and Klein, 1998; Shu et al., 2016). The Eph-ephrin signaling system promotes cell migration by regulating the reorganization of the actin cytoskeleton and increasing intercellular adhesiveness (Pasquale, 2008), suggesting that the common characteristics and molecular mechanisms of cancer cells can be modulated by them. Therefore, the Eph-ephrin complex can be used as a new diagnostic biomarker and potential molecular therapeutic target in cancers.

Ephrin ligands are divided in A-subclass (ephrin-A1-A5) and B subclass (ephrin-B1-B3) groups based on their sequence conservation. Ephrin-As are glycosyl phosphatidyl inositol (GPI)-anchored molecules and are usually bound by EphA receptors, while ephrin-Bs are transmembrane proteins with an extracellular binding domain for EphB receptors and cytoplasmic SAM/PDZ-binding motif (Kullander and Klein, 2002). Ephrin ligands have been extensively studied in morphogenesis and neural development. Recently, increasing attention has been given to its significance in the tumorigenesis and progression of various cancers. Substantial evidence indicates that ephrins play a vital role in tumor angiogenesis, invasion, metastasis, and tumor stemness maintenance (Lodola et al., 2017). Many ephrin ligands have been shown to be upregulated in multiple tumors and associated with poor prognosis, such as lung adenocarcinoma (Deng et al., 2021), breast cancer (Kaenel et al., 2012), colorectal cancer (Papadakos et al., 2022), prostate cancer (Zhao et al., 2021), bladder cancer (Mencucci et al., 2020), and other cancers (Surawska et al., 2004). Lin et al. (2021) reported that EFNA4 is highly expressed in cancer tissues and leads to poor prognosis in patients with HCC. In addition, recent studies have highlighted important roles of Eph-ephrin signaling in the tumor microenvironment (TME) and tumor immunity (Janes et al., 2021). The ephrin ligand members are widely expressed on diverse immune cell types and participate in regulating cell adhesion, migration, and activation of B and T lymphocytes (Jin et al., 2011; Mori et al., 2013). Moreover, they also recruit immunosuppressive cells such as myeloid-derived suppressor cells (MDSCs) and tumor-associated macrophages (TAMs) to the TME, inhibit the activity of cytotoxic T cells, and, thus, support tumor survival (Hanahan and Coussens, 2012). A recent study suggested that EFNA3 acts as an independent prognostic factor and correlates with immune cell infiltration in gastric cancer and lung adenocarcinoma (Deng et al., 2021; Zheng et al., 2021). However, the expression level and prognostic value of ephrin family genes and their association with tumor immunity have been less explored in HCC.

In this study, we performed a comprehensive analysis of ephrin family genes in HCC based on The Cancer Genome Atlas (TCGA), Genotype-Tissue Expression dataset (GTEx), Tumor Immune Evaluation Resource (TIMER) database, and some online bioinformatics analysis websites. We first explored the expression patterns of ephrin genes among 31 human cancer types. Then, the prognostic role of ephrin genes was discussed in HCC patients, and the association between prognosis-related ephrin genes and the TME, immune cell infiltration, immune subtypes, immune checkpoint biomarkers, gene mutation landscape, and drug response in HCC was further highlighted. Moreover, the differential expression of ephrins was validated in multiple HCC cell lines and 40 paired clinical tissue samples using RT–qPCR. The results of this study revealed the potential role of ephrin family genes as predictive biomarkers of prognosis and immunotherapy in patients with HCC, which warrants further in-depth study.

## Materials and methods

### Clinical tissue samples and ethics approval

In total, 40 paired fresh HCC tumorous and adjacent tissues were collected from the Second Affiliated Hospital of Nanchang University (Nanchang, China) between January 2021 and December 2021. The tissue samples were immediately frozen in liquid nitrogen after surgical resection and stored at −80°C until further analysis. The usage of tumor and adjacent normal tissues in this study was approved by The Second Affiliated Hospital of Nanchang University Medical Research Ethics Committee. All of the patients enrolled in this study provided written informed consent in accordance with the Helsinki Declaration and related guidelines.

### Public data acquisition and processing

RNA-seq data in the TPM (transcripts per million reads) format of pan-cancer datasets were downloaded from the UCSC XENA (https://xenabrowser.net/datapages/), which were processed by the Toil process (Vivian et al., 2017), and the samples were derived from the TCGA and GTEx datasets. All expression data were normalized on a log2 (TPM +1) scale. The cancer types with fewer than 3 samples were removed, and we ultimately obtained the expression data of 15,521 samples from 31 cancer types. Meanwhile, transcriptome profiling data of HCC projects harmonized to TPM were downloaded from TCGA (https://portal.gdc.cancer.gov/), including 374 tumor tissues and 50 normal samples. Furthermore, we also obtained clinical information and prognostic outcomes of HCC from the UCEC database, which was derived from a prognostic study of the TCGA dataset (Liu et al., 2018), including age, sex, histological grade, pathological stage, vascular invasion status, overall survival (OS), progression-free interval (PFI), and disease-specific survival (DSS).

### Expression patterns of ephrin genes in pan-cancer and their diagnostic value in HCC

Ephrin gene expression between tumor tissues and unpaired normal tissues in pan-cancer was analyzed and visualized using the Sangerbox online platform (http://sangerbox.com/) based on TCGA targeted GTEx datasets. The differential expression analysis of EFNs in HCC tissues compared with paired normal tissues was conducted in TCGA datasets by using the “limma” and “ggplot2” packages of R 4.0.5 software (http:///www.r-project.org/). The Wilcoxon rank sum test was applied for statistical analyses, and a value of *p* < 0.05 was considered to be statistically significant.

Receiver operating characteristic (ROC) curves and the area under the ROC curve (AUC) were employed to estimate the diagnostic ability of ephrin family genes, and “pROC” and “ggplot2” of R packages were used for visualization and analysis. An AUC of 0.5–0.7 indicates a lower level of diagnostic accuracy, an AUC of 0.7–0.9 suggests moderate accuracy, and an AUC above 0.9 indicates higher diagnostic accuracy.

Subsequently, we explored the correlation among ephrin genes at the mRNA expression level with Pearson’s correlation analysis, in which the “corrplot” R package was used to calculate the correlation coefficient (Pearson’s R), and Pearson’s R > 0.3 was considered statistically significant.

### Prognostic values and clinical feature correlation analyses of ephrin genes in HCC

First, we integrated the mRNA expression data of ephrin genes with clinical information based on the HCC project from the TCGA database. After removing the samples with incomplete follow-up information, the remaining patients were divided into high- and low-expression groups based on the best cutoff values of the expression of each ephrin gene. Kaplan-Meier analysis was performed to explore the relationship between EFN gene expression and prognostic indicators, including OS, PFI, and DSS. The “survminer” and “survival” R packages were used for statistical analysis and data visualization. The statistical significance was obtained with the log-rank test. In addition, independent prognostic factors for OS were identified by univariate and multivariate Cox regression analyses, integrating the following clinical features: age, sex, histological grade, and pathological stage. The results are presented as a hazard ratio (HR) and 95% confidence interval (CI), and statistical significance was defined as *p* < 0.05. The ephrin genes that significantly and independently affected OS were chosen for further analyses.

To further investigate the correlation between ephrin ligand genes and clinicopathological parameters, we compared the expression levels of ephrin genes with different clinical T stages, pathological stages, histological grades, and vascular invasion status in HCC patients. Student’s t test or one-way ANOVA was used to verify expression differences. Moreover, the binary logistic regression model was utilized to evaluate the association between ephrin gene expression and clinical characteristics, such as age (>60 vs. ≤ 60), sex (male vs. female), T stage (T3&T4 vs. T1&T2), N stage (N1 vs. N0), M stage (M1 vs. M0), pathological stage (stage III& IV vs. stage I&II), histological grade (G3&G4 vs. G1&G2), vascular invasion status (yes vs. no), AFP (ng/ml) (>400 vs. ≤400), and Child–Pugh grade (B&C vs. A). The patients were divided into high- or low-expression groups according to the median expression value of ephrin genes, and the expression grouping was used as the independent variable. The clinical characteristics were dependent variables, and the right factors in parentheses were used as references. The results are presented with odds ratios and *p value*s, and a *p value* of less than 0.05 (*p* < 0.05) was considered significant.

### Protein interaction and gene set enrichment analysis

The GeneMANIA online website (http://www.genemania.org) was applied to explore the interaction network of ephrin ligand members (EFNA3, EFNA4, and EFNB1), in which a large number of genomic and proteomic data were used to identify interactional genes with similar functions (Franz et al., 2018). The website mainly provides protein–protein interaction (PPI) predictions, including physical interaction, co-expression, co-localization, sharing of protein domains, genetic interactions, and signaling pathways. Furthermore, we also used the STRING database (https://string-db.org/) to clarify the interactive relationships among ephrin family genes and displayed the 50 most relevant proteins that interact with ephrin genes.

To investigate the biological role and uncover the potential biological mechanisms of ephrin genes in HCC, we conducted GSEA based on GSEA v.4.1.0 software (http://www.gsea-msigdb.org/gsea/index.jsp) and “c2. cp.kegg.v7.4. symbols.gmt,” which was downloaded from MSigDB (http://www.gseamsigdb.org/gsea/msigdb/collections.jsp). Gene sets with a *p value* < 0.05 and a false discovery rate (FDR) of q-value < 0.25 were considered significantly enriched pathways.

### Correlation of ephrin genes with the tumor microenvironment and tumor immunity

Previous studies have indicated that ephrin ligands and the Eph receptor signaling pathway significantly affect immune cell infiltration and change the tumor microenvironment (TME) (Yu et al., 2004; Yu et al., 2006; Lu et al., 2017). Thus, in this study, we explored the correlation between the expression of ephrin genes and TME and tumor immune cell infiltration. First, immune and stromal scores were calculated by the ESTIMATE algorithm using the “estimate” R package, which represents the infiltration levels of immune and stromal cells in different tumors, respectively. ESTIMATE scores are the sum of immune and stromal scores and show an inverse correlation with tumor purity. Then, Spearman correlation analysis was performed to analyze the correlation between the expression of ephrin ligand genes and immune scores, stromal scores, ESTIMATE scores, and tumor purity. The results are presented with scatterplots, and *p* < 0.05 was considered statistically significant.

Thereafter, the Tumor Immune Evaluation Resource (TIMER) database (http://timer.comp-genomics.org/), an online platform for comprehensive analysis of the specific gene(s) associated with tumor immune infiltrating cells (TIICs), was used to evaluate the association between the expression of ephrin family members and the infiltration levels of various immune cells in HCC samples. TIMER2.0 provides multiple immune infiltration estimations, including the TIMER, XCELL, QUANTISEQ, MCPCOUNTER, EPIC, CIBERSORT-ABS, and CIBERSORT algorithms. In this study, we selected the “Gene” module and used Spearman correlation analysis in TIMER 2.0, with a focus on exploring the association of ephrin genes with the infiltration levels of B cells, CD4^+^ T cells, CD8^+^ T cells, neutrophil cells, macrophage cells, and dendritic cells. A *p* value < 0.05 was considered statistically significant. The results are presented with scatterplots. In addition, we downloaded immune cell infiltration estimates for all TCGA tumor samples from the TIME2.0 database, which included immune cell infiltration levels in each HCC sample based on the XCELL, QUANTISEQ, MCPCOUNTER, EPIC, CIBERSORT-ABS, and CIBERSORT algorithms. We further integrated the immune cell infiltration data and ephrin gene expression to comprehensively analyze the correlation between ephrin gene expression and tumor immunity in HCC tissues by using the “scales,” “ggplot2,” and “ggtext” R packages. Spearman correlation coefficients were calculated to measure the strength of the statistical correlation between two variables. The results with *p* < 0.05 were considered significant and are presented with bubble plots.

Immune subtypes in cancers could effectively reflect intratumoral immune states. Six immune subtypes have been identified based on immune expression signatures and represent different immune functions, including C1 (wound healing), C2 (IFN-gamma dominant), C3 (inflammatory), C4 (lymphocyte depleted), C5 (immunologically quiet), and C6 (TGF-beta dominant) (Thorsson et al., 2018). To identify the relationship between the expression of ephrin genes and immune subtypes in HCC, we used the online TISIDB web portal (http://cis.hku.hk/TISIDB/) and the Kruskal–Wallis test to compare the expression of ephrin genes between different immune subtypes. *P* < 0.05 was considered statistically significant.

### Correlation analysis of ephrin family genes with immune checkpoint inhibitors (ICIs) biomarkers

Gene expression profiling within the tumor microenvironment could assess active innate and adaptive immune responses and may identify robust biomarkers for predicting the clinical benefit of checkpoint inhibitor strategies (Gibney et al., 2016). Thus, we utilized Spearman correlation analysis to assess the co-expression relationship between ephrin ligand genes and 47 immune checkpoint-related genes in HCC. The R packages “limma,” “reshape2” and “RColorBrewer” were used to conduct the co-expression analysis. The results are displayed with a heatmap. Furthermore, we thoroughly analyzed the expression connection between ephrin genes and four key immune-related genes: PDCD1 (PD-1), CTLA4, CD274 (PD-L1), and PDCD1LG2 (PD-L2). The results are presented with scatter plots, and a *p* value less than 0.05 (*p* < 0.05) indicated a significant correlation.

Tumor mutation burden (TMB) is defined as the total number of mutations per million bases detected in each tumor sample, including gene coding errors, base substitution, gene insertion or deletion errors. Microsatellite instability (MSI) is a hypermutator phenotype with hypermutability of short repetitive sequences in the genome and impaired DNA mismatch repair (MMR) in tumors (Cortes-Ciriano et al., 2017). Increasing studies have indicated that TMB and MSI are primary drivers of tumor immune responses and have been proven to be predictive biomarkers for ICIs (Lengyel, 2021; McGrail et al., 2021). In our study, we explored the correlation of ephrin family genes with TMB and MSI in HCC. First, gene mutation data in “varscan 2” format were downloaded from the TCGA database and then transformed to TMB data using Perl 5.30.0 software (https://www.perl.org/). MSI data of HCC patients were directly acquired from previous studies (Hause et al., 2016; Yang G. et al., 2019). Then, we compared EFN gene expression between the high- and low-TMB/MSI subgroups and further explored their association using Spearman correlation analysis. The “limma,” “ggpubr” and “reshape2” R packages were used for data analysis and visualization.

### Prediction of response to chemotherapy and targeted therapy

To date, chemotherapy and antiangiogenic targeted therapy are the main treatments for advanced HCC patients. Thus, we investigated the role of ephrin genes in predicting the sensitivity of HCC patients to chemotherapies and targeted drugs. In our study, six commonly used chemotherapeutic and targeted agents of HCC were selected, namely, camptothecin, cisplatin, doxorubicin, mitomycin C, gemcitabine, and sorafenib. First, the pRRophetic algorithm and “pRRophetic” R package were used to calculate the drug half-maximal inhibitory concentration (IC50) of common chemotherapy and targeted therapy drugs based on the Cancer Genome Project (CGP) cell lines data (Geeleher et al., 2014). Then, we compared the drug sensitivity of the six common drugs in HCC between the high- and low-expression subgroups of ephrin family members. Statistical significance was determined as a *p value* less than 0.05.

### Associations between the expression of ephrin family genes and mutational landscape genes

Single nucleotide polymorphisms (SNPs) refer to DNA sequence polymorphisms caused by variation in a single nucleotide at the genome level and widely exist in human genomic DNA. Abnormal SNPs promote the occurrence and development of tumors and contribute to treatment resistance. In our study, we analyzed the correlation between ephrin gene expression and SNPs in HCC. First, the format (MAF) file of somatic mutation information of HCC was obtained from the TCGA database, which was previously processed by the “varscan” method. The gene mutation frequency was calculated with the “maftools” R package. The top 15 genes with the highest mutation frequency were selected for comparison between the high- and low-expression groups of ephrin genes. We compared the genes and the mutational incidence rate between the two subgroups using the chi-squared test. *p* < 0.05 served as the significance threshold.

### Cell lines and cell culture

Five HCC cell lines (HCC-LM3, MHCC97-H, SMMC7721, Huh7, and HepG2) were purchased from Procell Life Science & Technology Co. Ltd. (Wuhan, China). The normal liver cell Line L02 was previously acquired from the Chinese Academy of Science. All cells were cultured in Dulbecco’s modified Eagle medium (DMEM; Solarbio, Beijing, China) supplemented with 10% FBS (Gibco, Grand Island, NY, United States), 100 µg/ml streptomycin and 100 U/ml penicillin sodium (Biotechnology, Beijing, China) in a humidified cell incubator containing 5% CO_2_ at 37°C. Subsequently, the mRNA levels of EFNs in each cell line were detected using real-time reverse transcription-quantitative polymerase chain reaction (RT–qPCR). The L-02 cell line served as a control.

### RNA extraction and RT–qPCR

Total RNA isolation from HCC cells and tissue samples was carried out by using TRIzol Reagent (Invitrogen, Carlsbad, CA, United States) according to the product manual. Subsequently, the RNA was reverse transcribed to complementary DNA (cDNA) using EasyScript® One-Step gDNA Removal and cDNA Synthesis SuperMix (AE311-03, TransGen Biotech, Beijing, China). Then, real-time quantitative PCR (qPCR) was performed using TB Green® Premix Ex Taq™ II (RR820A, TaKaRa, China). Glyceraldehyde 3-phosphate dehydrogenase (GAPDH) was used as the endogenous control. The relative gene expression of HCC cells was calculated according to the 2^−ΔΔCT^ method, and 2^−ΔCT^ was used to determine the mRNA expression in HCC tissues. qPCR assays were performed in triplicate. The gene primers for qPCR are listed in Table 1.

**TABLE 1:** Primers for RT-qPCR analysis targeting ephrin genes.

Gene name	Sequences (5′—3′)
EFNA1	F: TCA​GGC​CCA​TGA​CAA​TCC​AC; R: GTG​ACC​GAT​GCT​ATG​TAG​AAC​C
EFNA2	F: TAC​GCC​GTC​TAC​TGG​AAC​C; R: GAG​CCT​CGT​ACA​GGG​TCT​C
EFNA3	F: CAT​GCG​GTG​TAC​TGG​AAC​AG; R: AGA​TAG​TCG​TTC​ACG​TTC​ACC​T
EFNA4	F: CTC CGCCACGTAGTCTACTG; R: TAC​AAA​GCA​AAC​GTC​TCG​GGG
EFNA5	F: CGC​TAC​GCT​GTC​TAC​TGG​AAC; R: TTC​TGG​GAC​GGA​GTC​CTC​ATA
EFNB1	F: CGT​GTT​GGT​CAC​CTG​CAA​TAG; R: CAG​GCT​TCC​ATT​GGA​TGT​TGA
EFNB2	F: TAT​GCA​GAA​CTG​CGA​TTT​CCA​A; R: TGG​GTA​TAG​TAC​CAG​TCC​TTG​TC
EFNB3	F: CTC​GGC​GAA​TAA​GAG​GTT​CCA; R: GTG​AAG​CGG​AGA​TCC​AGG​TC
GAPDH	F: GGA​GCG​AGA​TCC​CTC​CAA​AAT; R: GGC​TGT​TGT​CAT​ACT​TCT​CAT​GG

qRT-PCR, quantitative real-time reverse transcription polymerase chain reaction.

F, forward primer; R, reverse primer.

### Statistical analysis

R software (https://www.r-project.org/, version 4.0.4) and Perl 5.30.0 software (https://www.perl.org/) were applied to conduct bioinformatics analyses. Student’s t test, one-way ANOVA, or the Wilcoxon rank sum test was used to assess the differences between groups. The log-rank test and Cox regression analysis were used for survival analysis. Spearman correlation was used in the correlation analyses. The differential gene expression in HCC cells or tissue samples was analyzed with Student’s t test or one-way ANOVA using GraphPad Prism 9.0 software (GraphPad Prism Software, Inc., La Jolla, CA, United States). All experiments were repeated in triplicate to calculate the mean ± standard deviation (SD). All statistical tests were two-sided, and statistical significance was set at *p* < 0.05.

## Results

### Ephrin family ligands are aberrantly expressed in pan-cancer

Based on the expression analysis of eight ephrin ligand genes (EFNA1, EFNA2, EFNA3, EFNA4, EFNA5, EFNB1, EFNB2, EFNB3) in 15,521 samples of 31 cancer types from TCGA and GTEx datasets, we found that EFNA1 expression was upregulated in 21 cancer types, including GBM, LGG, UCEC, BRCA, CESC, LUAD, ESCA, STES, COAD, PRAD, STAD, HNSC, KIRC, LIHC, BLCA, OV, PAAD, TGCT, ALL, LAML, and CHOL. In contrast, EFNA1 expression was downregulated in LUSC, WT, SKCM, THCA, and KICH **(** Supplementary Figure S1A **)**. The mRNA expression of EFNA2 in tumor tissues of GBM, LGG, UCEC, BRCA, CESC, LUAD, ESCA, STES, KIRP, COAD, PRAD, STAD, KIRC, LUSC, WT, SKCM, BLCA, THCA, OV, PAAD, TGCT, UCS, ALL, LAML, PCPG, and ACC was higher than that in corresponding normal tissues. Significant downregulation of EFNA2 was observed in LIHC, READ, and KICH (Supplementary Figure S1B). EFNA3 was upregulated in most cancer types, except for GBM, SKCM, and KICH **(** Supplementary Figure S1C). The expression level of EFNA4 was higher in 27 tumor tissues than in corresponding normal tissues, including GBM, LGG, UCEC, BRCA, CESC, LUAD, ESCA, STES, KIRP, COAD, PRAD, STAD, HNSC, LUSC, LIHC, WT, BLCA, THCA, READ, OV, PAAD, TGCT, UCS, ALL, LAML, ACC, and CHOL (Supplementary Figure S1D). EFNA5 gene expression was found to be upregulated in 13 tumor tissues but downregulated in 15 cancer types (Supplementary Figure S1E). EFNB1 was significantly distinctly expressed in 26 cancer types, with higher expression in cancer tissues observed in GBM, LGG, CESC, ESCA, STES, COAD, STAD, HNSC, LIHC, WT, OV, PAAD, UCS, ALL, LAML, and CHOL and lower expression observed in UCEC, BRCA, LUAD, KIRP, PRAD, KIRC, SKCM, THCA, PCPG, and KICH (Supplementary Figure S1F). EFNB2 was aberrantly expressed in 26 cancer types (Supplementary Figure S1G). The expression of EFNB3 was downregulated in multiple cancer types, including LIHC (Supplementary Figure S1H).

### The expression levels and diagnostic significance of ephrin family genes in HCC

**FIGURE 1:**
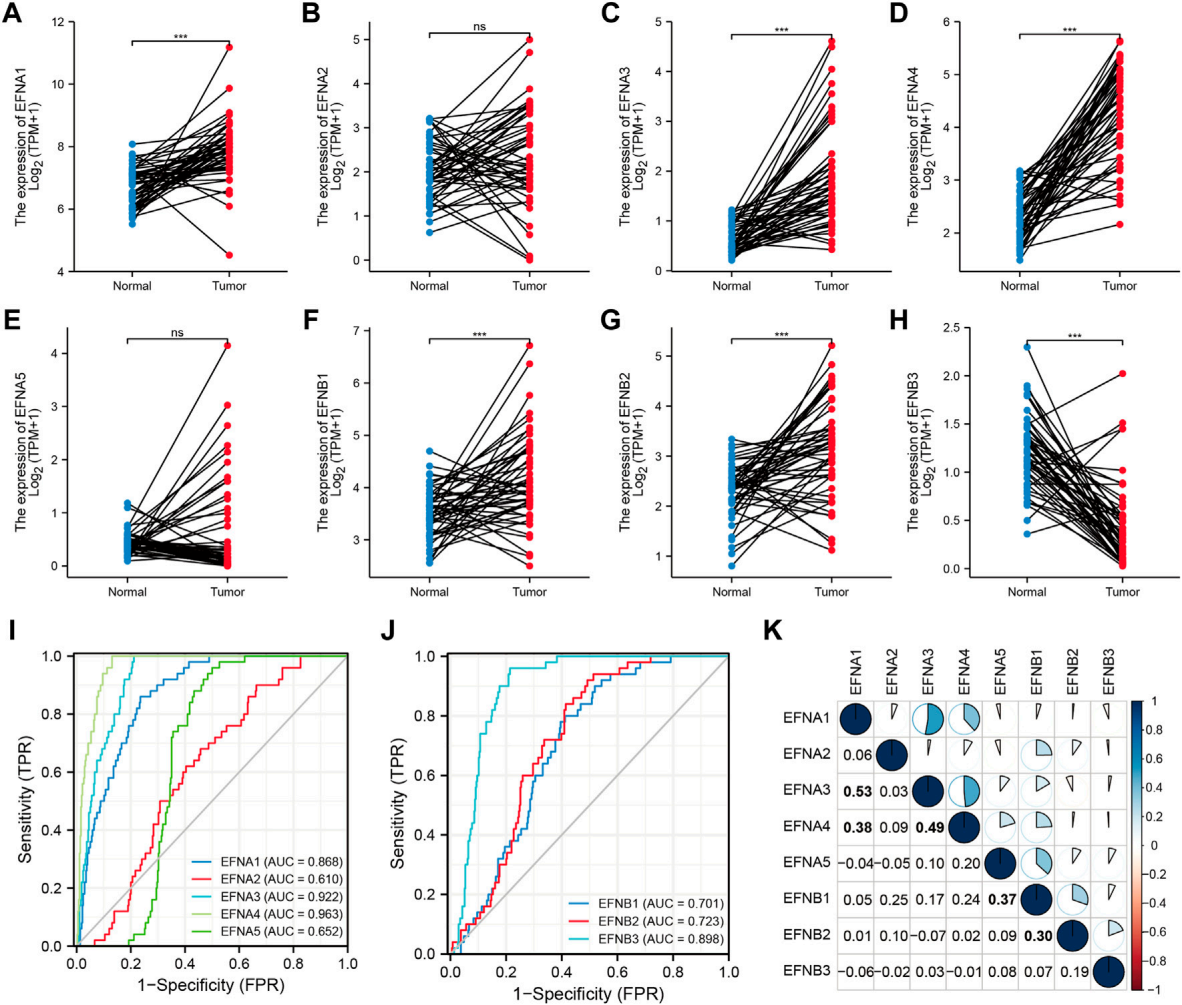
The expression levels and diagnostic significance of ephrin members in HCC tissues based on TCGA database. **(A–H)** The differential expression of EFNs (EFNA1, EFNA2, EFNA3, EFNA4, EFNA5, EFNB1, EFNB2, EFNB3) in tumor tissues compared with paired normal tissues in HCC. ns: no significance; ***p < 0.001; **(I,J)** The diagnostic role of EFNs identified by receiver operating characteristic (ROC) curves. **(K)** Correlation analysis of each EFN member based on Pearson’s correlation analysis. The bold values represent significant correlations between the EFN members.

Based on the TCGA datasets related to HCC, the mRNA expression levels of EFNs were detected in 50 paired tumor tissues and corresponding normal samples. As shown in Figures 1A–H, the expression of EFNA1, EFNA3, EFNA4, EFNB1, and EFNB2 was significantly increased in tumor tissues compared with normal tissues. EFNB3 expression was evidently decreased in tumor tissues, and no significant difference was observed in EFNA2 and EFNA5 expression between tumor and normal tissues. The differential expression results in paired HCC tissues of EFNA1, EFNA3, EFNA4, EFNB1, EFNB2, and EFNB3 coincided with the above pan-cancer analysis.

According to the expression levels of ephrin genes, we further evaluated the diagnostic accuracy of EFNs by calculating the AUC values of ROC curves. The results indicated that EFNA3 (AUC = 0.922) and EFNA4 (AUC = 0.963) showed higher diagnostic accuracy; EFNB1 (AUC = 0.701), EFNB2 (AUC = 0.723), and EFNB3 (AUC = 0.898) exhibited moderate diagnostic performance; and EFNA2 and EFNA5 exhibited a lower level of diagnostic accuracy, with an AUC <0.7 (Figures 1I,J).

Moreover, we also investigated the co-expression correlation among the eight ephrin genes using Pearson’s correlation analysis. The results (Figure 1K) showed that the expression of EFNA1 was positively correlated with EFNA3 (R = 0.53) and EFNA4 (R = 0.38); EFNA3 showed a positive correlation with EFNA4 (R = 0.49); EFNA5 expression was positively associated with EFNB1 (R = 0.37); and EFNB1 was related to EFNB2 expression (R = 0.3). However, the expression of EFNA2 and EFNB3 was not significantly associated with other ephrin genes.

### The association of ephrin genes with prognosis and clinical characteristics in HCC

**FIGURE 2:**
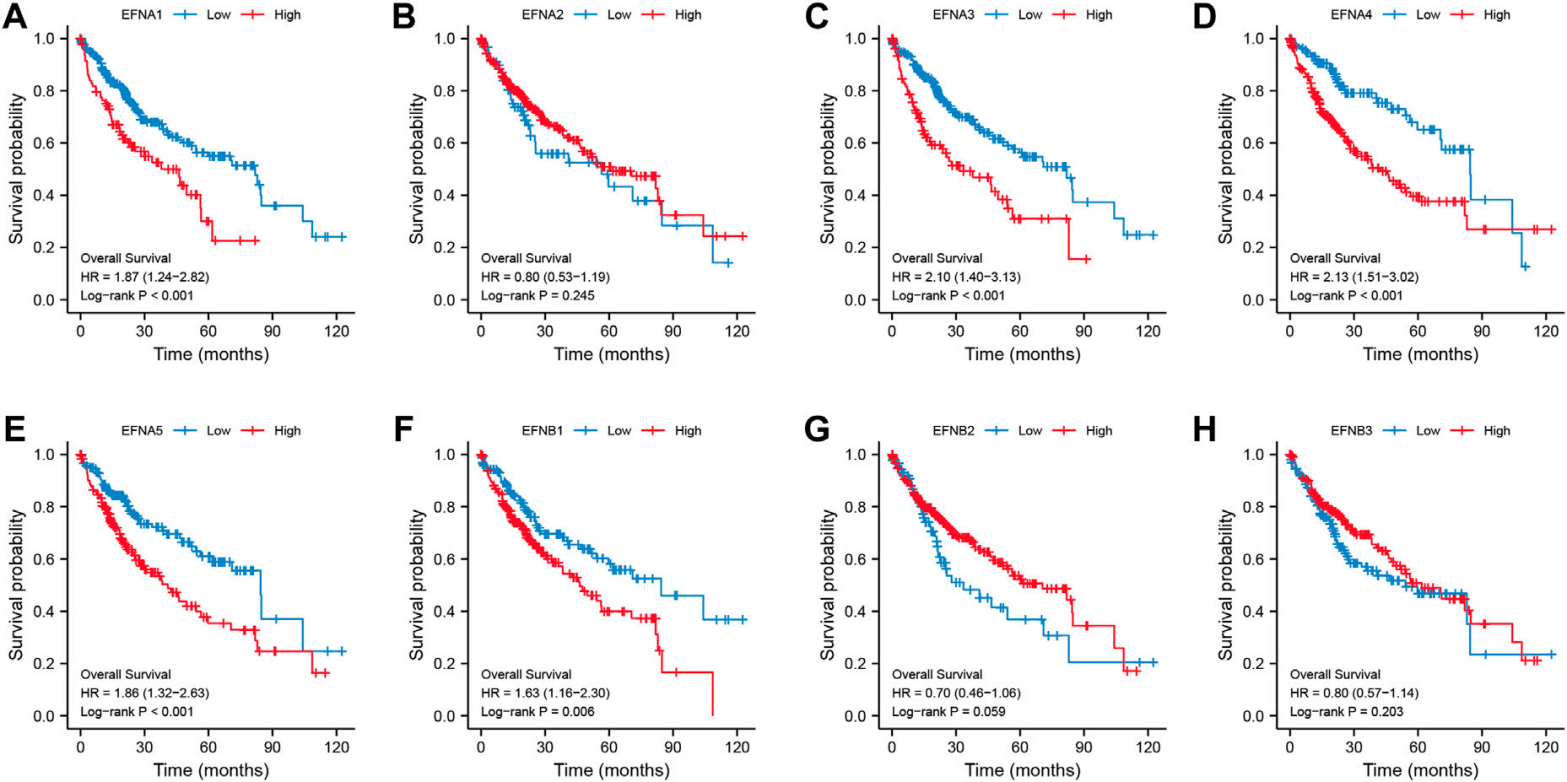
Correlation of ephrin genes expression with overall survival (OS) in patients with HCC based on Kaplan-Meier analysis **(A–H)**.

**FIGURE 3:**
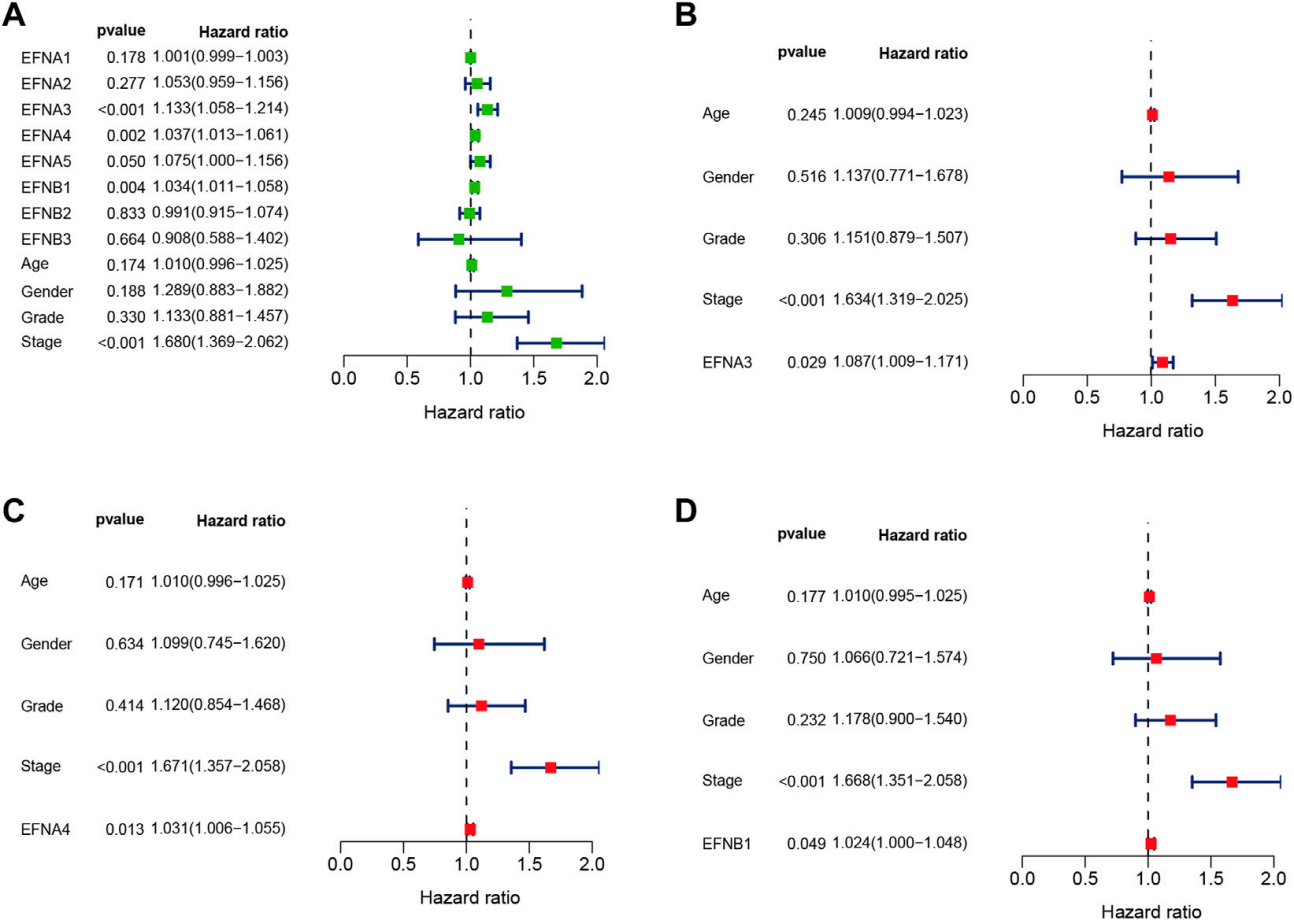
Univariate and multivariate Cox regression analyses of EFN members and clinicopathological parameters in HCC displayed with forest plots. **(A)** Univariate Cox regression analysis of EFNs and clinicopathological parameters. **(B–D)** Multivariate Cox regression analysis of ephrin members with significant prognostic significance.

We found that ephrin family genes were differentially expressed in patients with HCC. To further explore the prognostic influence of the eight EFNs on OS, PFI, and DSS in HCC patients, the Kaplan-Meier method and log-rank test were performed in patients with HCC. For OS (Figure 2), the results suggested that the patients in the high-expression groups of EFNA1 (*p* < 0.001), EFNA3 (*p* < 0.001), EFNA4 (*p* < 0.001), EFNA5 (*p* < 0.001), and EFNB1 (*p* = 0.006) showed worse OS than those in the low-expression groups, while there was no significant correlation between the expression of EFNA2, EFNB2, EFNB3 and OS. We next explored the effect of ephrin genes on PFI. As shown in Supplementary Figure S2A, higher EFNA3 and EFNA4 expression was related to shorter PFI (*p* = 0.006 and *p* = 0.008, respectively), while the opposite result was observed for EFNA2 (*p* = 0.047) and EFNB3 (*p* = 0.007); the expression of EFNA1, EFNA5, EFNB1, and EFNB2 was not significantly associated with PFI. The DSS results of Kaplan-Meier analysis indicated that the expression of EFNA1 (*p* = 0.002), EFNA3 (*p* = 0.001), EFNA4 (*p* = 0.001), EFNA5 (*p* = 0.027), and EFNB1 (*p* = 0.004) was negatively correlated with DSS in patients with HCC (Supplementary Figure S2B **)**. Subsequently, univariate and multivariate Cox regression analyses were performed to identify the prognostic factors for OS by integrating the EFN expression and clinical factors (age, sex, histological grade, and pathological stage). Univariate Cox analysis suggested that the expression of EFNA3, EFNA4, and EFNB1 and pathological stage were risk factors for OS (*p* < 0.05; Figure 3A). Remarkably, these factors were proven to be independent prognostic factors for OS in HCC based on multifactor Cox regression analysis (Figures 3B–D). In brief, these results showed that EFNA3, EFNA4, and EFNB1 could serve as effective prognostic predictors in patients with HCC. We therefore focused on EFNA3, EFNA4, and EFNB1 for our subsequent analysis.

**FIGURE 4:**
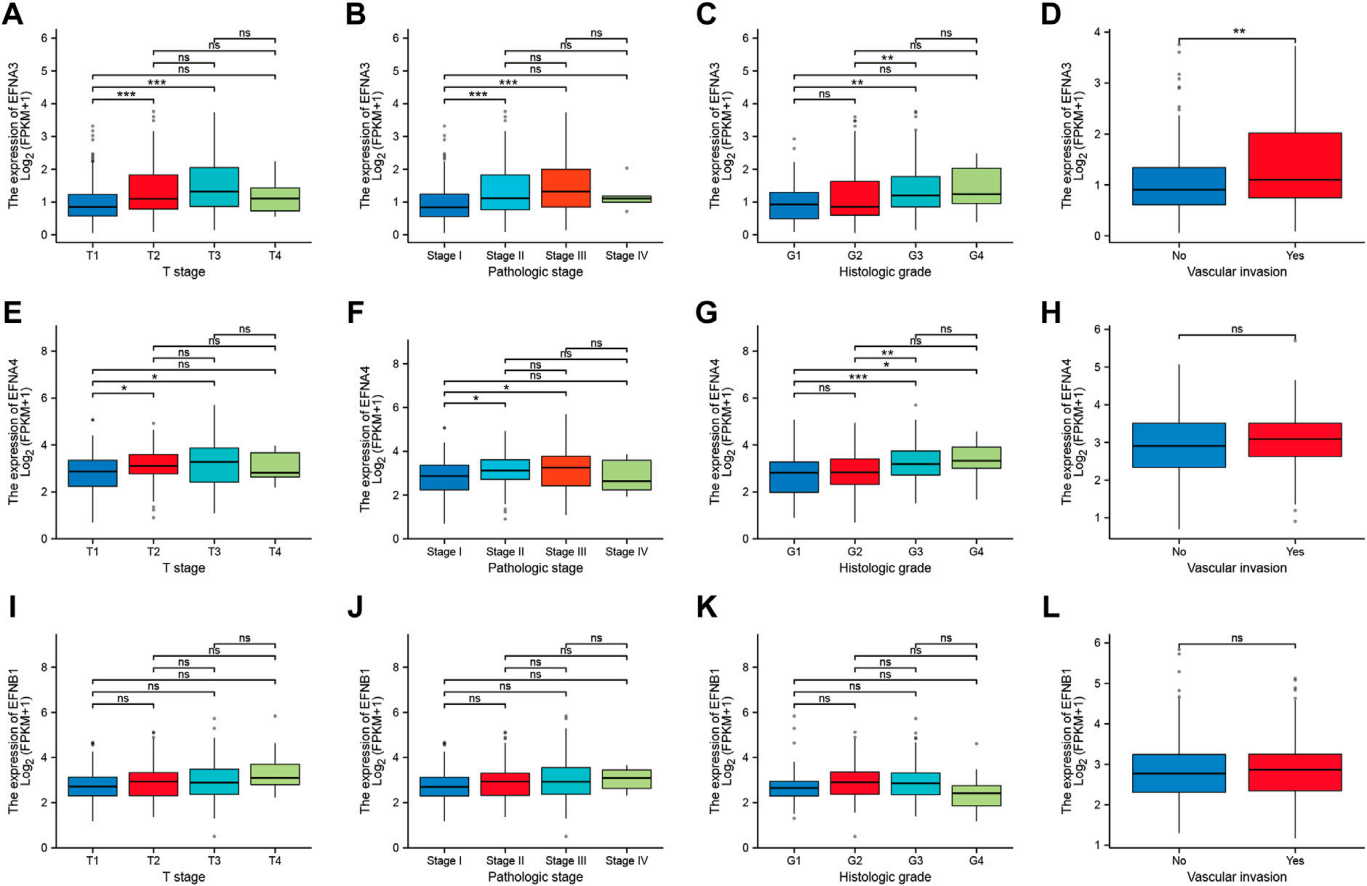
Correlation between prognosis-related ephrins and tumor stage, pathological stage, histological grade, and vascular invasion status in HCC. **(A–D)** The differential expression of EFNA3 associated with different tumor stages **(A)**, pathological stages **(B)**, histological grades **(C)**, and vascular invasion **(D)**. **(E–H)** The expression levels of EFNA4 for different tumor stages **(E)**, pathological stages **(F)**, histological grades **(G)**, and vascular invasion **(H)**. **(I–L)** The correlation between EFNB1 expression and tumor stage **(I)**, pathological stage **(J)**, histological grade **(K)**, and vascular invasion **(L)**. ns: no significance; *p < 0.05; **p < 0.01; ***p < 0.001.

To address how the ephrin genes affect survival outcomes, we further investigated the relationship between prognosis-related genes and clinicopathology features in HCC, including T stages, pathological stages, histological grades, and vascular invasion status. The results indicated that patients with more advanced T stages and pathological stages tended to have higher expression levels of EFNA3 and EFNA4 (Figures 4A,B,E,F). Similarly, the expression of EFNA3 and EFNA4 was positively correlated with histological grade (Figures 4C,G). We also found that patients with vascular invasion showed higher expression of EFNA3 (Figure 4D), but no significant difference was observed in EFNA4 (Figure 4H). However, there was no significant difference in EFNB1 expression among different T stages, pathological stages, histological grades, and vascular invasion statuses (Figures 4I–L). In addition, binary logistic regression analysis was used to explore the association between EFNs expression and different clinical characteristics. As shown in Table 2, we found that the patients in the high EFNA3 expression group exhibited a higher T stage (T3&T4 vs. T2&T1, *p* < 0.001) and pathological stage (Stage III& IV vs. Stage I& II*, p* < 0.001) and vascular invasion (Yes vs. No*, p* = 0.005) than those in the low EFNA3 expression group. The patients with high EFNA4 expression were associated with a higher histological grade (G3&G4 vs. G1&G2, *p* < 0.001) and AFP levels (>400 ng/ml vs. ≤400 ng/ml, *p* < 0.011). Similarly, high expression of EFNB1 tended to correlate with higher tumor size (T3&T4 vs. T1&T2, *p* = 0.034) and advanced TNM stage (Stages III & IV vs. Stages I & II, *p* = 0.028). However, the expression of EFNA3, EFNA4, and EFNB1 showed no significant difference between age subgroups (>60 vs. ≤60), sex subgroups (male vs. female), N stages (N1 vs. N0), M stages (M1 vs. M0), and Child–Pugh grades (B&C vs. A).

**TABLE 2:** The correlation between prognosis-related ephrin genes expression and clinicopathology characteristics.

Characteristics	Total(N)	EFNA3		EFNA4		EFNB1	
		Odds ratio	*p-*value	Odds ratio	*p-*value	Odds ratio	*p*-value
Age (>60 vs. ≤ 60)	373	0.73 (0.49–1.10)	0.132	0.93 (0.62–1.39)	0.719	0.80 (0.53–1.20)	0.275
Gender (Male vs. Female)	374	0.80 (0.52–1.24)	0.320	0.93 (0.60–1.43)	0.740	0.69 (0.45–1.07)	0.098
T stage (T3&T4 vs. T1&T2)	371	2.78 (1.70–4.63)	<0.001	1.44 (0.90–2.33)	0.128	1.67 (1.04–2.71)	0.034
N stage (N1 vs. N0)	258	2.64 (0.33–53.85)	0.402	2.83 (0.36–57.36)	0.373	NA	0.994
M stage (M1 vs. M0)	272	2.58 (0.33–52.59)	0.414	0.87 (0.10–7.37)	0.894	1.14 (0.14–9.65)	0.894
Pathologic stage (Stage III & IV vs. Stage I& II)	350	2.94 (1.78–4.98)	<0.001	1.41 (0.87–2.30)	0.161	1.72 (1.06–2.82)	0.028
Histologic grade (G3&G4 vs. G1&G2)	369	2.94 (1.90–4.60)	<0.001	2.37 (1.54–3.68)	<0.001	0.96 (0.63–1.47)	0.860
Vascular invasion (Yes vs. No)	318	1.97 (1.24–3.16)	0.005	1.51 (0.95–2.41)	0.082	1.46 (0.92–2.33)	0.111
AFP (ng/ml) (>400 vs. ≤ 400)	280	1.53 (0.88–2.70)	0.137	2.10 (1.19–3.78)	0.011	0.89 (0.50–1.55)	0.674
Child-Pugh grade (B&C vs. A)	241	0.50 (0.18–1.23)	0.144	0.61 (0.24–1.48)	0.285	0.60 (0.22–1.48)	0.282

### Protein interactions and gene set enrichment analysis of prognosis-related ephrin genes in HCC

**FIGURE 5:**
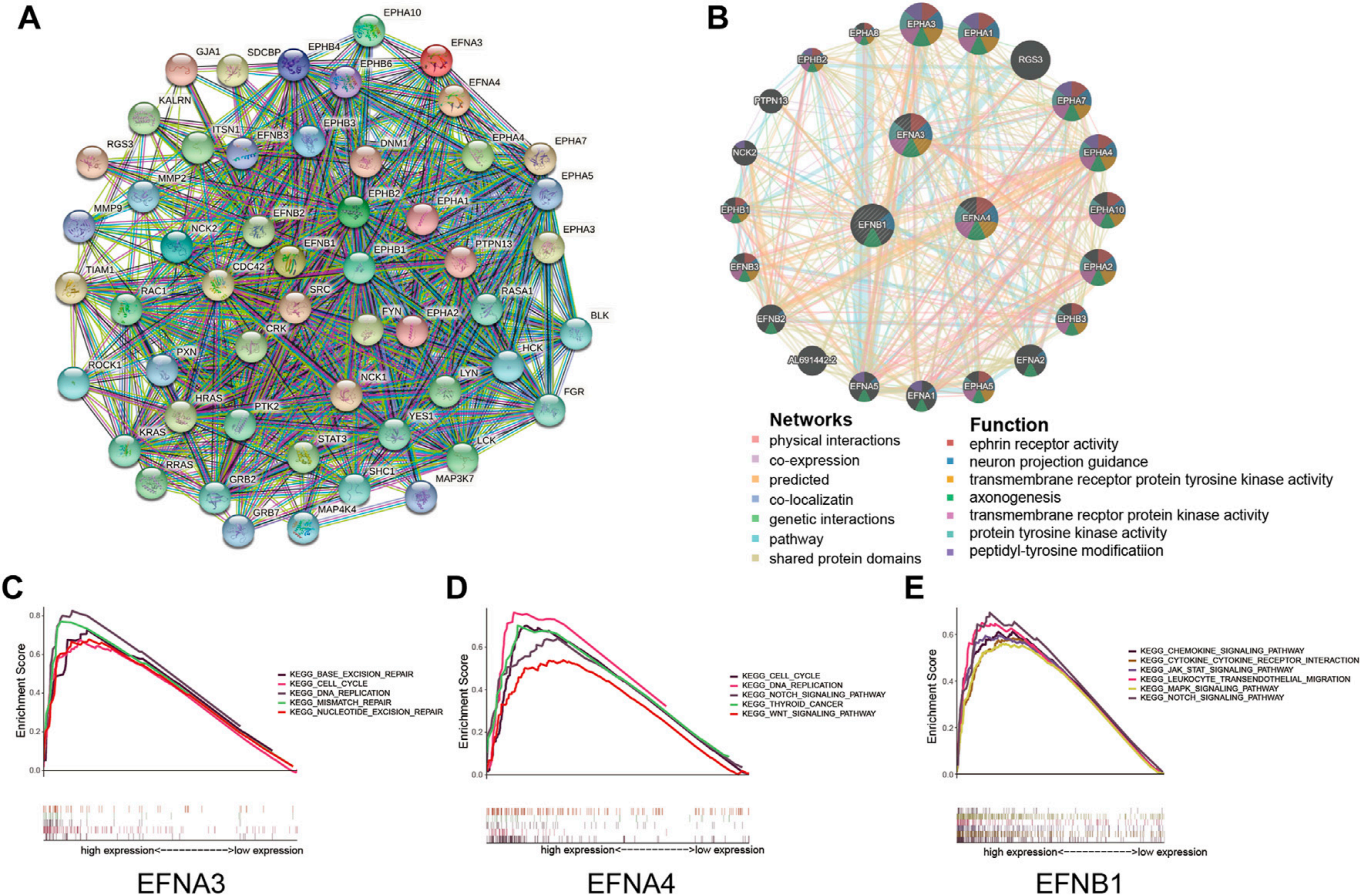
Protein interactions and gene set enrichment analysis (GSEA) of prognosis-related ephrin members (EFNA3, EFNA4, and EFNB1). **(A)** Protein–protein interactions (PPIs) based on the STRING database. **(B)** Interaction network for ephrin members based on the GeneMANIA database. **(C–E)** Gene set enrichment analysis (GSEA) of prognosis-related ephrin members, including EFNA3 **(C)**, EFNA4 **(D)**, and EFNB1 **(E)**.

To explore the interactional proteins of prognosis-related ephrin genes (EFNA3, EFNA4, and EFNB1), protein–protein interaction networks were constructed by using STRING. The network diagram in Figure 5A shows the 50 proteins most correlated with EFNA3, EFNA4, and EFNB1 and their interaction network based on the STRING database. GeneMANIA is available to explore gene interactions, and the results displayed the top 20 genes with the most relevance to EFNA3, EFNA4, and EFNB1 in accordance with physical interactions, co-expression, co-location, genetic interaction, pathway, and shared protein domains (Figure 5B). We found that both in the gene and protein levels, the prognosis-related EFNs were mainly associated with Eph receptors, such as EPHA3, EPHA1, EPHA4, EPHA10, and EPHA2. Gene function prediction suggested that these genes were mostly involved in ephrin receptor activity, neuron projection guidance, protein kinase activity, axonogenesis, and peptidyl-tyrosine modification (Figure 5B).

Furthermore, we conducted GSEA to further investigate the potential biological mechanisms of ephrin genes in HCC. The results suggested that high expression of EFNA3 was positively related to 43 gene sets at *p* value < 0.05 and FDR <0.25. The most significant pathways enriched in the high EFNA3 group were “cell cycle,” “DNA replication,” “base excision repair,” “mismatch repair,” and “nucleotide excision repair” (Figure 5C). High expression of EFNA4 was distinctly positively correlated with the “cell cycle,” “DNA replication,” “thyroid cancer,” “NOTCH signaling pathway,” and “WNT signaling pathway” (Figure 5D). The GSEA results of EFNB1 showed that “JAK/STAT signaling pathway,” “MAPK signaling pathway,” “NOTCH signaling pathway,” “chemokine signaling pathway,” “chemokine and chemokine receptor interaction,” and “leukocyte trans-endothelial migration” were enriched in the EFNB1 high-expression group (Figure 5E).

### Ephrin family members are correlated with TME and tumor immunity in HCC

**FIGURE 6:**
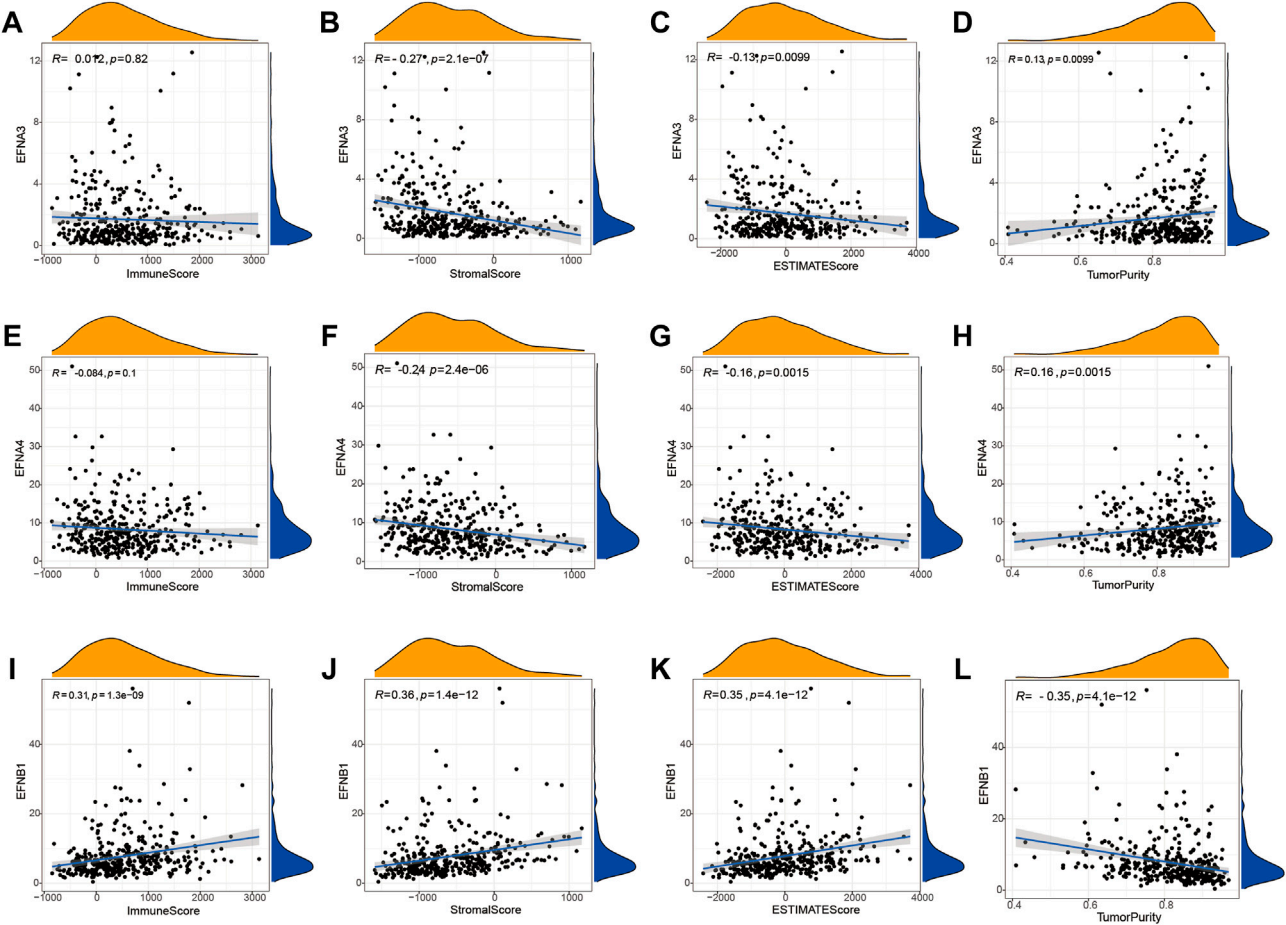
Correlation between the expression of prognostic ephrin genes and the tumor microenvironment based on the ESTIMATE algorithm. **(A–D)** The correlation between EFNA3 expression and immune/stromal/ESTIMATE/tumor purity scores. **(E–H)** The correlation between EFNA4 expression and immune/stromal/ESTIMATE/tumor purity scores. **(I–L)** The correlation between EFNB1 expression and immune/stromal/ESTIMATE/tumor purity scores. R: Spearman’s rank coefficient.

To further discuss the potential correlation between ephrin genes and the tumor immune microenvironment, we applied the ESTIMATE algorithm to calculate immune and stromal scores for each HCC sample and then analyzed the association of ephrin genes (EFNA3, EFNA4, and EFNB1) with immune scores, stromal scores, ESTIMATE scores, and tumor purity by using the Spearman correlation method. As shown in Figure 6, the expression of EFNA3 was evidently negatively related to stromal scores (R = −0.27, *p* = 2.1e-07) and ESTIMAT scores (R = −0.13, *p* = 0.0099) but positively related to tumor purity (R = 0.13, *p* = 0.0099) in HCC (Figures 6A–D). Analogously, EFNA4 was negatively correlated with stromal scores (R = −0.24, *p* = 2.4e-06) and ESTIMAT scores (R = −0.16, *p* = 0.0015) but positively correlated with tumor purity (R = 0.16, *p* = 0.0015) (Figures 6E–H). The immune scores (R = 0.31, *p* = 1.3e-09), stromal scores (R = 0.36, *p* = 1.4e-12), and ESITIMAT scores (R = 0.35, *p* = 4.1e-12) showed a significantly positive correlation with EFNB1 expression, while EFNB1 expression was negatively correlated with tumor purity (R = −0.35, *p* = 4.1e-12) in HCC (Figures 6I–L).

**FIGURE 7:**
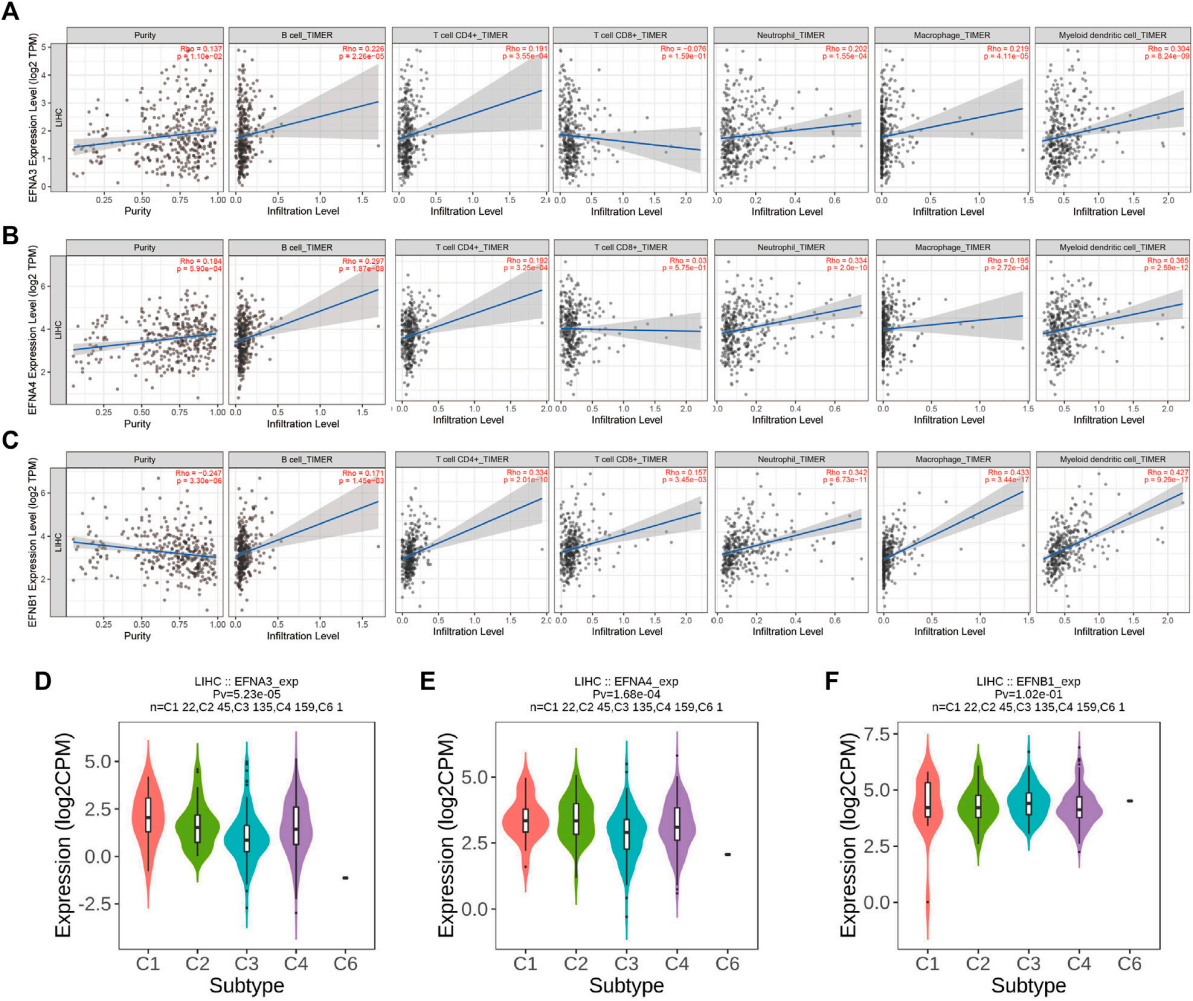
Correlation of significant prognostic ephrin genes with tumor-infiltrating immune cells and immune subtypes in HCC using TIMER algorithms. **(A)** EFNA3; **(B)** EFNA4; and **(C)** EFNB1. Tumor purity is shown in the panels on the left. **(D–F)** The correlation between prognostic ephrin genes (EFNA3, EFNA4, and EFNB1) expression and immune subtypes in HCC using TISIDB. Rho, Spearman’s rank coefficient; Pv, *p* value; C1, wound healing; C2, IFN-gamma dominant; C3, inflammatory; C4, lymphocyte depleted; C5, immunologically quiet; C6, TGF-b dominant.

Moreover, the TIMER database and Spearman correlation analysis were used to explore the correlation of ephrin family genes (EFNA3, EFNA4, EFNB1) with the infiltration levels of immune cells in HCC by using TIMER algorithms. The results indicated that EFNA3 was notably positively associated with tumor purity (Rho = 0.137, *p* = 1.1e-02), B cells (Rho = 0.226, *p* = 2.26e-05), CD4^+^ T cells (Rho = 0.191, *p* = 3.55e-04), neutrophil cells (Rho = 0.202, *p* = 1.55e-04), macrophage cells (Rho = 0.219, *p* = 4.11e-05), and dendritic cells (Rho = 0.304, *p* = 8.24e-09) in HCC (Figure 7A). The expression of EFNA4 showed positive associations with tumor purity (Rho = 0.184, *p* = 5.9e-04), B cells (Rho = 0.297, *p* = 1.87e-08), CD4^+^ T cells (Rho = 0.192, *p* = 3.25e-04), neutrophil cells (Rho = 0.334, *p* = 2.0e-10), macrophage cells (Rho = 0.195, *p* = 2.72e-04), and dendritic cells (Rho = 0.385, *p* = 2.59e-12) (Figure 7B). However, no correlation was observed between EFNA3/EFNA4 expression and the infiltration levels of CD8^+^ T cells (Figures 7A,B). EFNB1 was found to have a statistically significant negative correlation with tumor purity (Rho = −0.247, *p* = 3.3e-06) and a positive correlation with B cells (Rho = 0.171, *p* = 1.45e-03), CD4^+^ T cells (Rho = 0.334, *p* = 2.02e-10), CD8^+^ T cells (Rho = 0.157, *p* = 3.45e-03), neutrophil cells (Rho = 0.342, *p* = 6.73e-11), macrophage cells (Rho = 0.433, *p* = 3.44e-17), and dendritic cells (Rho = 0.427, *p* = 9.29e-17) (Figure 7C). In addition, we applied other algorithms, such as XCELL, QUANTISEQ, MCPCOUNTER, EPIC, CIBERSORT-ABS, and CIBERSORT, to comprehensively analyze the association of prognostic ephrin genes with tumor immunity. The results showed that EFNA3 expression was evidently correlated with most immune cells, such as hematopoietic stem cells (XCELL, Rho = −0.394, *p* = 3.06E-15), endothelial cells (XCELL, Rho = −0.39, *p* = 6.36E-15), and common lymphoid progenitors (XCELL, Rho = 0.311, *p* = 8.53E-10) (Supplementary Figure S3; Supplementary Table S1). Similarly, there were significant associations between EFNA4 expression and various immune cells (Supplementary Figure S4; Supplementary Table S2). We also found that the expression of EFNB1 was positively related to most immune and stromal cells in HCC tissues, such as cancer-associated fibroblasts, myeloid dendritic cells, and M2 macrophages (Supplementary Figure S5; Supplementary Table S3).

In addition, we further investigated the potential relevance between ephrin family genes (EFNA3, EFNA4, EFNB1) and different immune subtypes of HCC, and the results revealed that the expression of EFNA3 and EFNA4 was prominently correlated with immune subtype (*p* = 5.23e-05, *p* = 1.68e-04, respectively). EFNA3 and EFNA4 were highly expressed in the C1 subtype but expressed at low levels in the C3 subtype (Figures 7D,E). This finding indicated that EFNA3 and EFNA4 may be more involved in wound healing but less involved in inflammatory processes. However, no significant association was observed between EFNB1 expression and immune subtype (Figure 7F).

### Relationship between ephrin gene expression and ICIs

**FIGURE 8:**
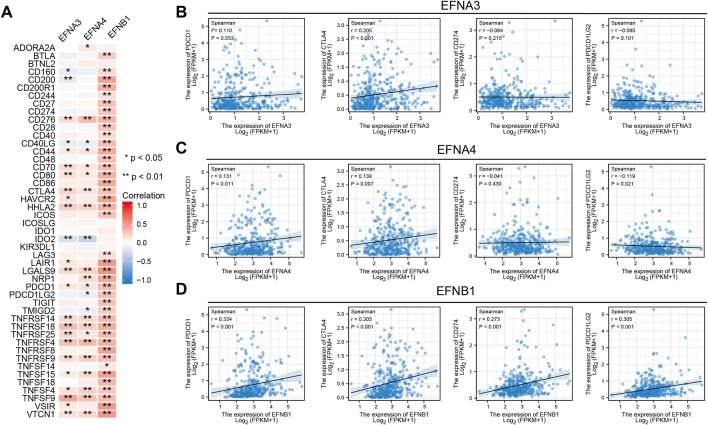
Relationship between significant prognostic ephrin members and immune checkpoint-related genes in HCC. **(A)** Heatmap displaying the coexpression relationship between prognosis-related ephrins and 47 immune checkpoint-related genes. **(B–D)** Scatterplots displaying the association of EFNA3 **(B)**, EFNA4 **(C)**, and EFNB1 **(D)** expression with four key immune checkpoint-related genes (PDCD1, CTLA4, CD274, and PDCD1LG2) with Spearman correlation analysis. r: Spearman’s rank coefficient; *p < 0.05; **p < 0.01.

It was reported that immune checkpoint-related genes, TMB, and MSI can serve as effective predictors for ICIs. Thus, we assessed the latent correlations of prognosis-related ephrin genes (EFNA3, EFNA4, EFNB1) with these ICIs biomarkers in HCC. A multigene correlation heatmap of gene co-expression analyses showed that EFNA3 expression was significantly related to 24 immune checkpoint-related genes, EFNA4 was significantly correlated with 23 immune-related genes, and there was a highly positive correlation between EFNB1 expression and immune-related genes (Figure 8A). We highlighted the association between ephrin genes expression and four key immune checkpoint-related genes (PDCD1, CTLA4, CD274, and PDCD1LG2) using Spearman correlation analysis. The scatter plots showed that the expression of PDCD1 and CTLA4 was positively correlated with EFNA3 expression (r = 0.11, *p* = 0.033; r = 0.205, *p* < 0.001), while no association was found between EFNA3, CD274 and PDCD1LG2 (Figure 8B). EFNA4 exhibited a significant positive correlation with PDCD1 (r = 0.131, *p* = 0.011) and CTLA4 (r = 0.139, *p* = 0.007) but a negative correlation with PDCD1LG2 (r = –0.119, *p* = 0.021) **(** Figure 8C **)**. Remarkably, EFNB1 expression was significantly positively associated with PDCD1 (r = 0.334, *p* < 0.001), CTLA4 (r = 0.305, *p* < 0.001), CD274 (r = 0.273, *p* < 0.001), and PDCD1LG2 (r = 0.305, *p* < 0.001) (Figure 8D).

**FIGURE 9:**
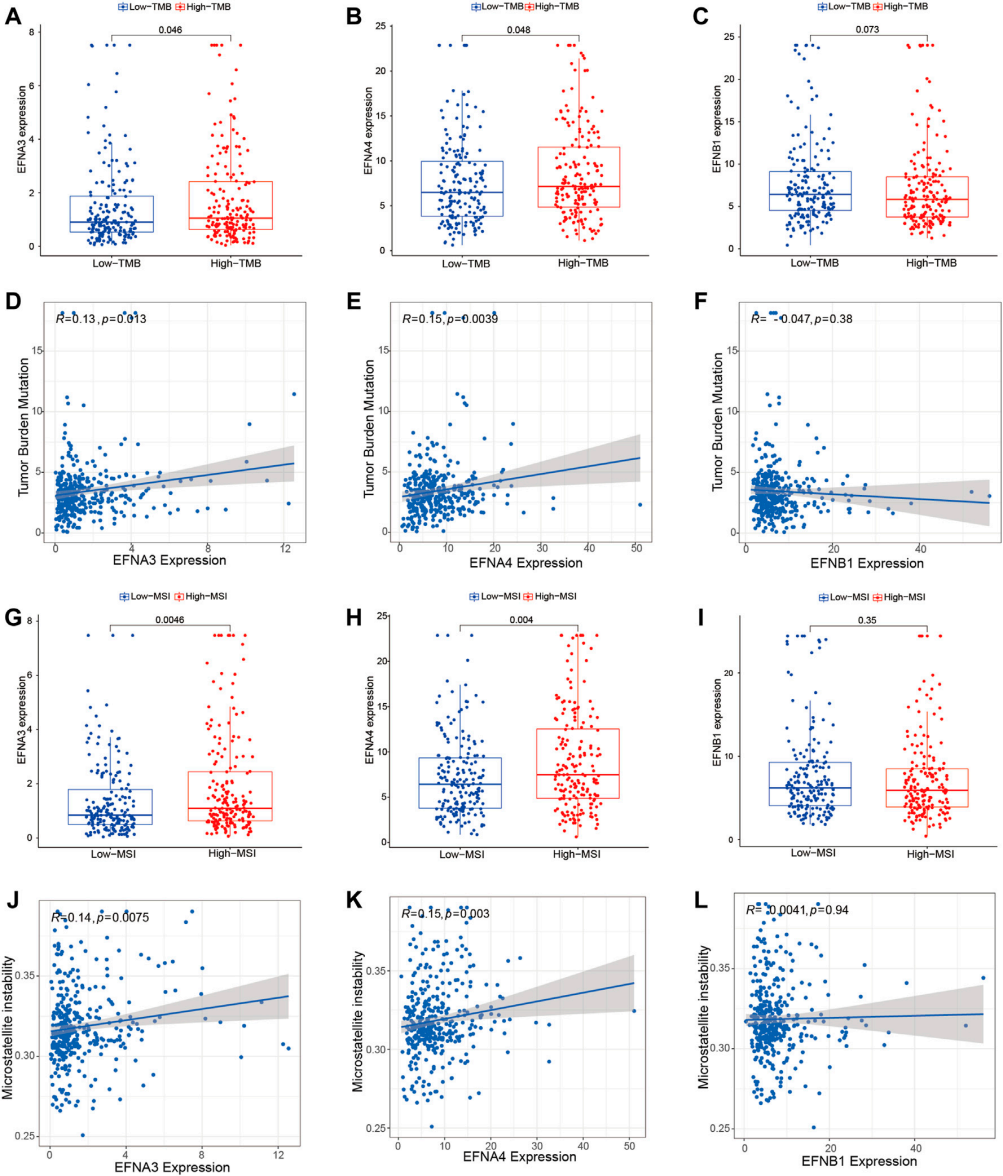
Association of prognosis-related ephrins with tumor mutation burden (TMB) and microsatellite instability (MSI) in HCC. **(A–C)** The differential expression of EFNA3, EFNA4, and EFNB1 in the low- and high-TMB groups. **(D–F)** Scatterplots displaying the association between TMB scores and EFNA3 expression **(D)**, EFNA4 expression **(E)**, and EFNB1 expression **(F)**. **(G–I)** The differential expression of EFNA3, EFNA4, and EFNB1 in the low- and high-MSI groups. **(J–L)** Scatterplots displaying the association between MSI scores and EFNA3 expression **(J)**, EFNA4 expression **(K)**, and EFNB1 expression **(L)**. r: Spearman’s rank coefficient.

Furthermore, we performed an investigation to analyze the association of ephrin genes with TMB and MSI by integrating gene expression and TMB/MSI data. We found that HCC patients with a high TMB highly expressed EFNA3 (*p* = 0.046) and EFNA4 (*p* = 0.048) (Figures 9A,B), and Spearman correlation analysis also indicated that TMB levels were positively correlated with the expression of EFNA3 (R = 0.13, *p* = 0.015) and EFNA4 (R = 0.15, *p* = 0.0039) (Figures 9D,E). However, there was no significant association between EFNB1 expression and TMB scores (Figures 9C,F). Similarly, HCC patients with high MSI exhibited higher EFNA3 (*p* = 0.0046) and EFNA4 (*p* = 0.004) expression than those with low MSI (Figures 9G,H), and the expression levels of EFNA3 (R = 0.14, *p* = 0.0075) and EFNA4 (R = 0.15, *p* = 0.003) were significantly positively related to MSI scores based on Spearman correlation analysis (Figures 9J,K). No significant relationship was observed between EFNB1 expression and MSI status (Figures 9I,L).

### Ephrin genes predict the response to chemotherapy and targeted therapy in HCC

**FIGURE 10:**
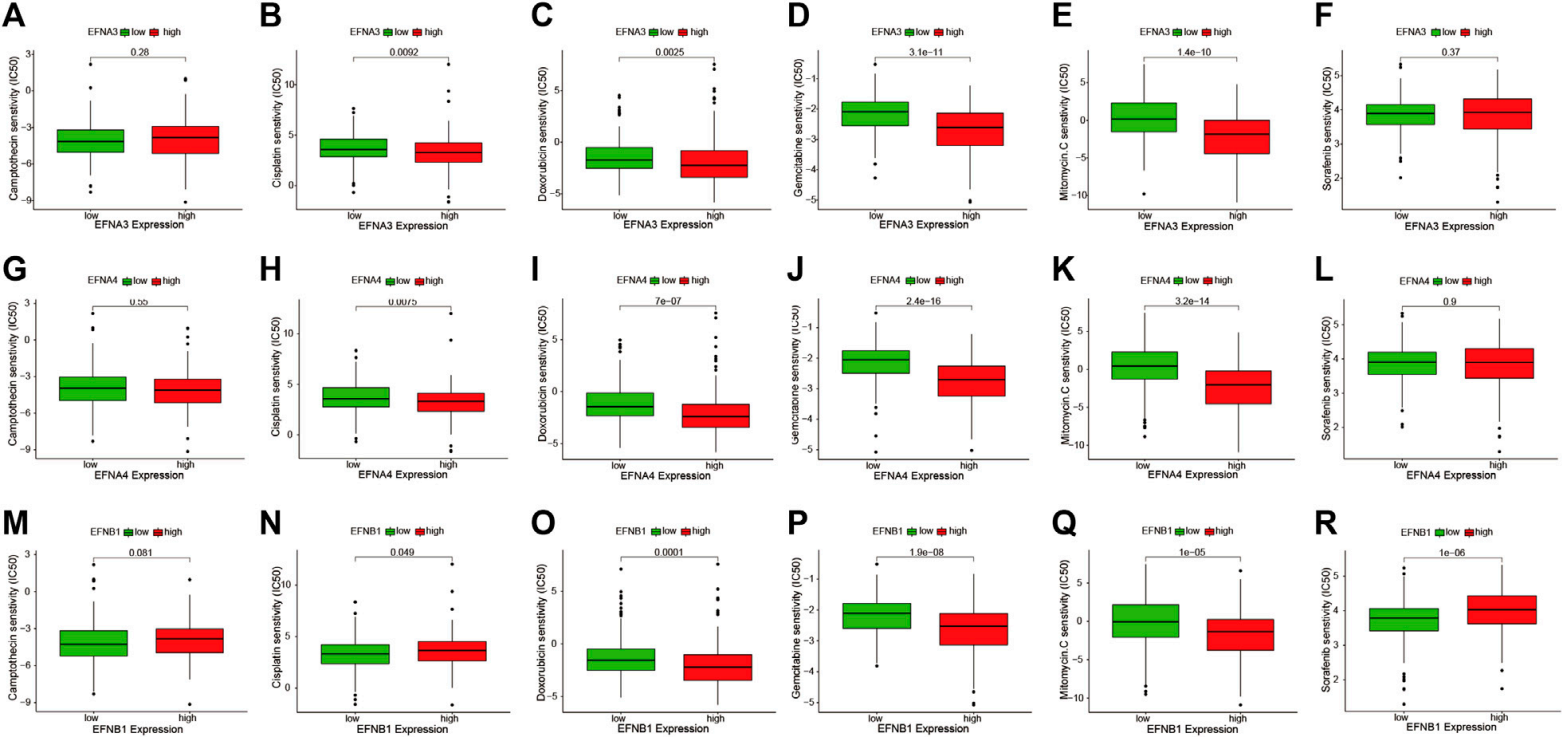
The IC50 values of six commonly used drugs (camptothecin, cisplatin, doxorubicin, gemcitabine, mitomycin C, and sorafenib) were compared between high- and low-expression subgroups of prognostic ephrin genes in HCC. **(A–F)** EFNA3, **(G–L)** EFNA4, and **(M–R)** EFNB1.

To probe the correlation between prognosis-related ephrin genes (EFNA3, EFNA4, and EFNB1) and drug sensitivity to chemotherapy and targeted therapy, we compared the IC50 values of six commonly used drugs (camptothecin, cisplatin, gemcitabine, doxorubicin, mitomycin C, and sorafenib) in the high- and low-EFNs expression subgroups using pRRophetic algorithm. As shown in Figures 10A–R, a lower IC50 of cisplatin (*p* = 0.0092), doxorubicin (*p* = 0.0025), gemcitabine (*p* = 3.1e-11), and mitomycin C (*p* = 1.4e-10) was present in the high EFNA3 expression group compared with the low expression group, indicating that HCC patients with high EFNA3 expression appeared to be more susceptible to these drugs. However, no significant difference was observed between camptothecin and sorafenib (Figures 10A–F). The expression of EFNA4 was also significantly related to the IC50 of cisplatin (*p* = 0.0075), doxorubicin (p = 7e-07), gemcitabine (*p* = 2.4e-16), and mitomycin C (*p* = 3.2e-14), showing that the high-expression populations were more sensitive to these drugs, but the IC50 of camptothecin and sorafenib was not evidently different in the high- and low-expression groups (Figures 10G–L). Regarding the correlation between EFNB1 and drug sensitivity, we found that HCC patients with high EFNB1 expression exhibited a better drug response to doxorubicin (*p* = 0.0001), gemcitabine (*p* = 1.91e-8), and mitomycin C (*p* = 1e-05) than those with low EFNB1 expression, while the opposite results were discovered for cisplatin (*p* = 0.049) and sorafenib (*p* = 1e-06) (Figures 10M–R). In brief, the results indicated that EFNs expression may contribute to evaluating the response to chemotherapy and targeted therapy in patients with HCC. Regrettably, the IC50 of immune checkpoint inhibitors is currently not available in GDC cell lines, thus we could not predict the response to ICIs by using “pRRophetic” R package.

### Correlation between ephrin gene expression and gene mutational landscape

In the HCC project of TCGA database, a total of 369 samples were included for detecting genetic mutations. Then, we integrated the gene expression and mutation data and further compared gene mutational frequency in high- and low-expression groups of ephrin genes (EFNA3, EFNA4, and EFNB1). The results are highlighted in Supplementary Figure S6. The top 15 genes with the highest mutational frequency are presented in the Waterfall plots. The mutational frequency of the 15 genes showed significant differences in the high- and low-expression groups of EFNA3, including TP53 (*p* = 8.6e-04), ABCA13 (*p* = 5.4e-04), RB1 (*p* = 0.04), DCHS2 (*p* = 0.03), HELZ (*p* = 0.04), DOCK10 (*p* = 0.04), MICAL3 (*p* = 0.02), COL3A1 (*p* = 0.02), ITGAD (*p* = 0.02), DENND4A (*p* = 0.02), CHSY3 (*p* = 0.04), ADGRB1 (*p* = 0.04), FAM65B (*p* = 0.04), BNC2 (*p* = 0.04), and FAM205A (*p* = 0.04) (Supplementary Figure S6A). With regard to EFNA4, we found that gene mutations were more common in the high expression group compared with the low expression group, such as TP53 (*p* = 8.6e-04), CTNNB1 (*p* = 5.3e-03), MUC4 (*p* = 0.04), RYR2 (*p* = 0.02), HMCN1 (*p* = 0.04), PREX2 (*p* = 0.03), MUC5B (*p* = 0.01), TDRD5 (*p* = 8.7e-03), SVEP1 (*p* = 0.04), ROBO1 (*p* = 0.03), EP300 (*p* = 0.04), and ARFGEF3 (*p* = 0.02), while higher mutation of IL6ST, DMBT1, and DOCK8 was observed in low EFNA4 expression group (Supplementary Figure S6B). The association between EFNB1 expression and the gene mutational landscape indicated that the mutational frequency of the ten mutated genes was higher in the high EFNB1 expression group, while the other five gene mutations occurred more commonly in the low expression group (Supplementary Figure S6C). In brief, it can be concluded that high expression of EFNA3, EFNA4, and EFNB1 may be relevant to more gene mutations and, thus, drive oncogenesis and tumor progression of HCC.

### Expression levels of ephrin genes in HCC cells and clinical tissues were identified by qPCR

**FIGURE 11:**
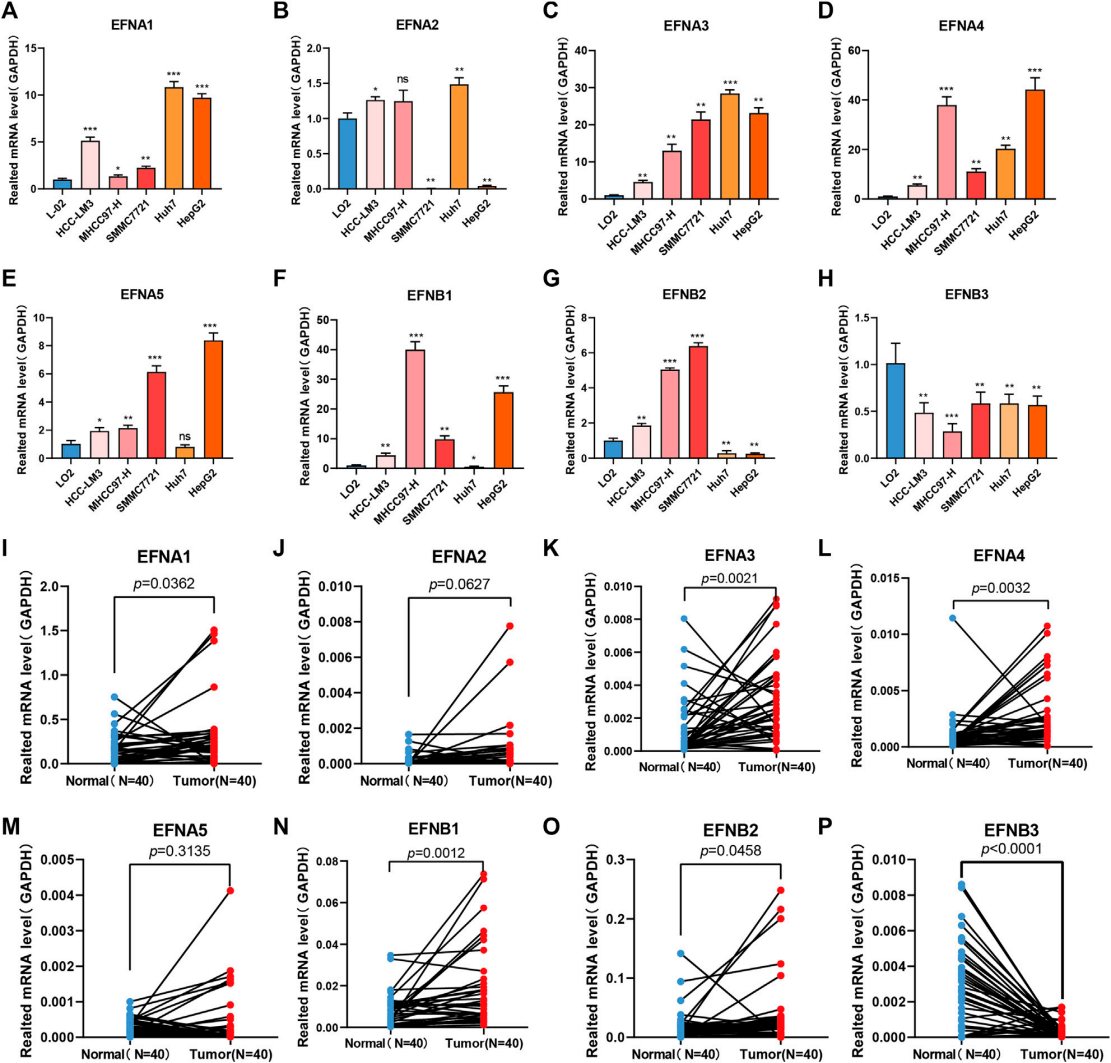
Ephrin family genes are abnormally expressed in HCC cell lines and HCC tissues. **(A–H)** RT–qPCR analysis of the mRNA expression of ephrin genes in five HCC cell lines (HCC-LM3, MHCC97-H, SMMC 7721, Huh-7, and HepG2) and a normal liver cell line (LO2). EFNA1 **(A)**, EFNA2 **(B)**, EFNA3 **(C)**, EFNA4 **(D)**, EFNA5 **(E)**, EFNB1 **(F)**, EFNB2 **(G)**, and EFNB3 **(H)**. **(I–P)** The mRNA expression of ephrin genes in 40 pairs of HCC tissues and adjacent para-carcinoma tissues was evaluated using qPCR. EFNA1 **(I)**, EFNA1 **(I)**, EFNA2 **(J)**, EFNA3 **(K)**, EFNA4 **(L)**, EFNA5 **(M)**, EFNB1 **(N)**, EFNB2 **(O)**, and EFNB3 **(P)**. GAPDH was used as an internal control. Error bars represent the means ± SEM (triplicate experiments). *p < 0.05; **p < 0.01; ***p < 0.001.

In the above bioinformatics analysis based on the TCGA dataset, we found that some ephrin genes (EFNA1, EFNA3, EFNA4, EFNB1, EFNB2) were significantly upregulated in HCC tissues, and EFNB3 was downregulated in HCC tissues, while EFNA2 and EFNA5 showed no significant differences between cancer tissues and adjacent normal tissues. To validate the results of the bioinformatics analysis, RT-qPCR was applied to detect mRNA expression in five HCC cell lines (HCC-LM3, MHCC97-H, SMMC 7721, Huh-7, and HepG2) and 40 paired HCC tissues. The results suggested that the mRNA expression of EFNA1, EFNA3 and EFNA4 was evidently higher in five HCC cell lines (HCC-LM3, MHCC97-H, SMMC 7721, Huh-7, and HepG2) (*p* < 0.05) (Figures 11A,C,D) and tumor tissues (*p* = 0.0362, *p* = 0.0021 and *p* = 0.0032, respectively) (Figures 11I,K,L) than in a normal liver cell line (L-02) and paired para-cancerous tissues. EFNA2 and EFNA5 were highly expressed in certain cell lines (Figures 11B,E) and showed no significant differences between cancer tissues and adjacent normal tissues (Figures 11J,M). The expression of EFNB1 was significantly increased in HCC-LM3, MHCC97-H, SMMC 7721, and HepG2 cells compared with the normal liver cell line (L-02), while EFNB1 expression was decreased in Huh-7 cells compared with the L-02 control (*p* < 0.05) (Figure 11F). Similarly, EFNB1 expression was significantly higher in cancer tissues than in paired adjacent normal tissues (*p* = 0.0012) (Figure 11N). The expression of EFNB2 was obviously higher in HCC cell lines (HCC-LM3, MHCC 97-H, SMMC 7721) but lower in Huh7 and HepG2 compared to the expression in normal liver cell line (L-02) (Figure 11G). Moreover, EFNB2 had a higher expression level in cancer tissues than in para-carcinoma tissues (*p* = 0.0458) (Figure 11O). In addition, the level of EFNB3 in the HCC cells and cancerous tissues was significantly reduced compared with the normal liver cell line L-02 (*p*<0.05) (Figure 11H) and the para-carcinoma tissues (*p* < 0.0001) (Figure 11P). These experimental results were consistent with those of the bioinformatics analysis.

## Discussion

The Eph/ephrin bidirectional signaling system is composed of a family of tyrosine kinase receptors and their plasma membrane-bound ligands (ephrins), which act as vital regulators for a variety of physiological and biological activities, such as axon guidance, cell–cell interactions, cell migration, and angiogenesis. Recently, an increasing number of studies have focused on its role in tumorigenesis and metastatic potential as related to tumor growth and survival. Aberrant ephrin expression is closely correlated with tumorigenicity, tumor vasculature, invasion, and metastasis in many types of human cancers, including HCC (McCarron et al., 2010). Thus, our study emphasized exploring the expression pattern, prognostic value and potential function of ephrin family genes in HCC, which may play a crucial role in the discovery of novel inhibition targets and therapeutic strategies for patients with HCC.

The ephrin ligands are aberrantly expressed in a variety of tumors and have been implicated in tumor progression, malignancy, and prognosis (Ieguchi and Maru, 2019). In HCC, for example, the expression level of EFNA1 is positively related to microscopic portal invasion after curative resection (Wada et al., 2014). EFNA2 was significantly upregulated in HCC cell lines and tissue samples, and its overexpression was associated with more aggressive tumor behaviors (Feng et al., 2010). EFNA3 was upregulated in HCC tissues, and its overexpression was associated with more aggressive tumor behaviors (Husain et al., 2022). EFNA4 is highly expressed and leads to poor prognosis in patients with HCC (Lin et al., 2021). The expression of EFNB1 is significantly higher in HCC tissues than in nontumor tissues and contributes to tumor progression *in vivo* by promoting neovascularization in HCC (Sawai et al., 2003). Although ephrin family genes have been extensively studied, the role of ephrins in cancers is not yet understood, as some tumors present with elevated levels of ephrin expression, while others demonstrate decreased expression (McCarron et al., 2010). In our study, the expression levels of ephrin members were comprehensively analyzed in 31 human cancer types based on TCGA and GTEx datasets. We found that the expression of EFNA1, EFNA2, EFNA3, EFNA4, EFNB1, and EFNB2 was upregulated in the tumor tissues of most cancers compared with corresponding normal tissues. EFNA5 and EFNB1 showed low expression in most cancers. In addition, this study focused on investigating the expression levels of ephrin genes and their relationship to prognosis in HCC. We found that the expression of EFNA1, EFNA3, EFNA4, EFNB1, and EFNB2 was significantly higher in HCC tissues than in paired normal tissues, and higher expression of EFNA1, EFNA3, EFNA4, EFNA5, and EFNB1 was associated with worse overall survival in patients with HCC. Whereas EFNB3 showed low expression in cancerous tissues, EFNA2 and EFNA5 expression showed no evident difference between tumor and normal tissues. Moreover, we have validated this differential expression results of bioinformatic analysis *via* performing RT-qPCR in HCC cell lines and clinical tissue samples. Besides, all the ephrin genes except EFNA2 and EFNA5 presented high disease diagnostic performance for HCC, with AUC>0.7. Cox regression analysis indicated that EFNA3, EFNA4, and EFNB1 were independent prognostic factors for OS and were defined as prognosis-related ephrin genes. We also discovered a significant correlation between the expression of EFNA3, EFNA4, and EFNB1 and T stage, pathological stage, histological grade, and vascular invasion. In short, these findings suggest that some ephrin genes (EFNA3, EFNA4, and EFNB1) are closely related to malignant biological behavior, such as tumor growth, vascular invasion and distant metastasis, and, thus, could be used as promising diagnostic and prognostic biomarkers in patients with HCC.

It has been reported that ephrins are abnormally expressed in multiple tumors and implicated in tumor development and metastasis, but their specific mechanism is still unclear. In this study, we focused on analyzing the protein–protein correlation and potential biological mechanisms of prognostic ephrin genes (EFNA3, EFNA4, and EFNB1) in HCC. The PPI network indicated that the three ephrins were mainly associated with Eph receptors and participated in ephrin receptor activity, protein kinase activity, neuron projection guidance, axonogenesis, and peptidyl-tyrosine modification, which was consistent with the results reported in previous work (McCarron et al., 2010). Furthermore, the potential biological mechanisms of EFNA3, EFNA4, and EFNB1 in HCC exhibit large variation based on GSEA. High EFNA3 expression was mainly involved in the following pathways: “cell cycle,” “DNA replication,” “base excision repair,” “mismatch repair,” and “nucleotide excision repair.” EFNA4 may affect tumor progression by changing pathways such as the “cell cycle,” “DNA replication,” “thyroid cancer,” “NOTCH signaling pathway,” and “WNT signaling pathway.” EFNB1 mainly participates in cancer-related pathways, such as the “JAK/STAT signaling pathway,” “MAPK signaling pathway,” and “NOTCH signaling pathway,” as well as immune regulation processes, including the “chemokine signaling pathway,” “chemokine and chemokine receptor interaction,” and “leukocyte transendothelial migration.” A previous study reported that suppression of EFNA3 expression promotes cell proliferation, migration, and invasion and regulates EMT in oral squamous cell carcinoma via the PI3K/AKT signaling pathway (Wang et al., 2020). In contrast, EFNA3 contributes to tumor cell self-renewal, proliferation and migration in HCC under hypoxia via SREBP1/ACLY-mediated metabolic rewiring in HCC (Husain et al., 2022). EFNA4 influences the proliferation and migration of HCC cells by promoting EphA2 phosphorylation at Ser897, activating the PIK3R2/GSK3β/β-catenin signaling pathway loop (Lin et al., 2021). A novel anti-EFNA4 drug (PF-06647263) binds specifically to EFNA4-expressing cells and subsequently induces DNA cleavage and apoptosis/cell death in triple-negative breast and ovarian tumors (Damelin et al., 2015; Garrido-Laguna et al., 2019). In general, during the process of tumor progression, ephrins may play critical roles through different mechanisms, which can vary among different genes or cancer types. Therefore, further experiments are needed to elucidate the specific molecular mechanisms of prognosis-related ehprins in HCC.

The tumor microenvironment has crucial roles in the development and progression of HCC, and distinct immune features, such as inflamed and noninflamed classes of HCC, and different genomic signatures are correlated with the immune therapy response (Llovet et al., 2022). Emerging evidence indicates that the Eph/ephrin signaling system plays a pivotal role in remodeling the tumor microenvironment and regulating immune cell infiltration (Janes et al., 2021). Unique microenvironments caused by cancer cells in turn induce the abnormal expression of the Eph/ephrin complex (Iwasaki et al., 2018; Husain et al., 2022). For example, EFNB1, which is widely expressed on T cells, B cells, and monocytes/macrophages, has been proven to mediate various immune events, such as lymphocyte activation and adhesion, T-cell differentiation and survival, regulation of acquired immune responses, and cytokine production (Yu et al., 2004; Luo et al., 2011). EFNB1 and two Eph receptors (EPHB6 and EPHB4) collaborate to repulsively control follicular T-helper cell retention in the germinal center and promote interleukin 21 (IL-21) production by T cells locally (Lu et al., 2017). All previous studies are consistent with the results of functional enrichment analysis in our study showing that EFNB1 is closely involved in immune regulation. However, the function of other EFNs in the tumor immune response is limited. In our study, we systematically analyzed the correlation between EFNs expression and TME scores, tumor immune cell infiltration, and immune subtypes using different immune algorithms. Our results suggest that EFNA3 and EFNA4 were negatively related to stromal and ESTIMATE scores but positively associated with tumor purity in HCC, which is consistent with the results obtained by Deng et al. (2021) in lung adenocarcinoma that EFNA3 is negatively associated with immunity and stromal infiltration. Moreover, EFNA3 and EFNA4 were positively associated with immune cell infiltration of B cells, CD4^+^ T cells, neutrophils, macrophages, and DCs but were not related to CD8^+^ T cells. We also discovered that EFNB1 was positively correlated with immune, stromal, and ESTIMATE scores but negatively correlated with tumor purity. Furthermore, we found a significant positive association between EFNB1 and different immune response cells toward cancer, such as B cells, CD4^+^ T cells, CD8^+^ T cells, neutrophils, macrophages, and DCs. These findings revealed that high expression of EFNA3, EFNA4, and EFNB1 in HCC tissues is not only related to tumor progression and poor prognosis but also promotes immune cell infiltration, which may improve antitumor immune responses.

In the past decade, antitumor responses have achieved unprecedented rates of long-lasting tumor responses in patients with a variety of cancers, including HCC, which can be realized by antibodies blocking the CTLA-4 or PD-1 pathway, either alone or in combination (Ribas and Wolchok, 2018). In HCC, tremelimumab plus durvalumab yields superior overall survival versus sorafenib (Kelley et al., 2021). The combination of atezolizumab and bevacizumab improves overall survival relative to sorafenib, which has already gained FDA approval for use in patients with HCC (Qin et al., 2021). Despite these major advances, more than half of HCC patients still do not respond to ICIs. Moreover, no reliable predictive biomarker of response to immunotherapy is available to guide personalized treatment and improve survival. Several potential biomarkers, such as PD-L1 expression, TMB, and specific genomic alterations, have been proposed based on exploratory end points in HCC trials (Pinter et al., 2021). The combined PD-L1 positivity score was associated with response to pembrolizumab and PFS in patients with HCC (Zhu et al., 2018). Patients with higher MSI and TMB may be more sensitive to ICIs based on previous studies in non-small-cell lung cancer and colon cancer (Samstein et al., 2019; Schrock et al., 2019). However, TMB is generally low and MSI is rare in HCC, which may limit their utility as biomarkers to predict ICI outcomes. Based on the current evidence, the incorporation of several predictive factors, such as genetic, TMB, MSI, and microenvironmental factors, may be more likely to estimate the response to ICIs than a single biomarker. Therefore, we performed a comprehensive correlation analysis between EFNs expression and previous biomarkers of ICIs, including immune checkpoint-related genes, TMB, and MSI. The results indicated that EFNA3 and EFNA4 were significantly related to some immune-related genes, TMB, and MSI in HCC; EFNB1 was positively associated with most immune-related genes, such as PD-1, CTLA4, PD-L1, and PD-L2, but unrelated to TMB and MSI scores. These findings suggest that EFNs may be used as integrated biomarkers to predict the therapeutic efficacy of ICIs in HCC. Nevertheless, studies on immunotherapy are still far from mature, especially in the aspect of sensitivity to ICIs, and the IC50 of ICIs has not been included in GDC database, which restricted our analyses to immunotherapy sensitivity *via* EFNs genes expression.

In this study, we found the expression levels of prognosis-related ephrin genes (EFNA3, EFNA4, and EFNB1) were associated with certain drugs sensitivity to chemotherapy and targeted therapy, the patients with higher expression of EFNA3, EFNA4, and EFNB1 may be more susceptible to these drugs. However, the high EFNA3/EFNA4 expression associated with worse overall survival in patients with HCC. How to explain this discrepancy? Firstly, our study is a retrospective data based on a public database, the treatment drugs for these patients was not available in TGCA database, which means the patient with high EFNs expression and poor prognosis probably did not use sensitive drugs. Secondly, in the drugs sensitivity analysis, we used pRRophetic algorithm to compared the IC50 values of common drugs in the high- and low-EFNs expression subgroups, which was based on expression matrix and drug information of the Cancer Genome Project (CGP) cell lines. The clinical roles of this analysis may guide drugs selection and predict drugs response in certain EFNs expression populations. Furthermore, the EFNs expression is associated to other prognostic factors, such as gene mutational landscape and tumor immune microenvironment. In brief, the patient with high EFNs expression exhibiting a better response to certain drugs does not mean a better prognosis.

In summary, we conducted comprehensive analyses of ephrin family members in HCC to explore their expression patterns and prognostic values using multiple databases. We discovered that EFNA3, EFNA4, and EFNB1 were highly expressed in HCC tissues compared with normal samples, and the high expression of these genes was associated with tumor progression and vascular invasion and, thus, led to poor prognosis in patients with HCC. Moreover, we found that prognosis-related EFNs were closely related to the TME, immune cell infiltration, immune subtypes, and biomarkers of ICIs, which may provide a new direction for the discovery of novel therapeutic targets and predictive biomarkers for immunotherapy.

## Data Availability

The original contributions presented in the study are included in the article/Supplementary Material, further inquiries can be directed to the corresponding author.

## Supplementary material

SUPPLEMENTARY FIGURE S1 The expression levels of ephrin family genes in 31 cancer types based on TCGA and GTEx datasets . **(A–H)** **p* < 0.05; ***p* <0.01; ****p* < 0.001.

SUPPLEMENTARY FIGURE S2 Association of ephrin expression with progression-free interval (PFI) **(A)** and disease-specific survival (DSS) **(B)** in HCC based on Kaplan–Meier analysis.

SUPPLEMENTARY FIGURE S3 Correlation between EFNA3 and tumor-infiltrating immune cells in HCC based on the XCELL, QUANTISEQ, MCPCOUNTER, EPIC, CIBERSORT-ABS, and CIBERSORT algorithms.

SUPPLEMENTARY FIGURE S4 Correlation between EFNA4 and tumor-infiltrating immune cells in HCC based on the XCELL, QUANTISEQ, MCPCOUNTER, EPIC, CIBERSORT-ABS, and CIBERSORT algorithms.

SUPPLEMENTARY FIGURE S5 Correlation between EFNB1 and tumor-infiltrating immune cells in HCC based on the XCELL, QUANTISEQ, MCPCOUNTER, EPIC, CIBERSORT-ABS, and CIBERSORT algorithms.

SUPPLEMENTARY FIGURE S6 Correlation between prognosis-related ephrin gene expression and gene mutational landscapes. **(A)** EFNA3; **(B)** EFNA4; and **(C)** EFNB1.

## Glossary

HCC: hepatocellular carcinoma
Eph: Erythropoietin-producing hepatocellular carcinoma
Ephrins: Eph receptor interacting ligands
EFNs: Eph receptor interacting ligands
TME: tumor microenvironment
TCGA: the Cancer Genome Atlas database
GTEx: Genotype-Tissue Expression database
TIMER: Tumor Immune Evaluation Resource
TPM: transcripts per million reads
TMB: tumor mutation burden
MSI: microsatellite instability
OS: Overall Survival
DSS: Disease-Specific Survival
PFI: Progression Free Interval
ROC: Receiver Operating Characteristics
AUC: Area Under the ROC Curve
PPI: protein-protein interaction
GSEA: gene set enrichment analysis
ICIs: immune checkpoint inhibitors
SNP: Single nucleotide polymorphisms
RT-qPCR: real-time reverse transcription-quantitative polymerase chain reaction
qPCR: Quantitative Real-time Polymerase Chain Reaction
ACC: Adrenocortical carcinoma
BLCA: Bladder urothelial carcinoma
BRCA: Breast invasive carcinoma
CESC: Cervical squamous cell carcinoma and endocervical adenocarcinoma
CHOL: Cholangiocarcinoma
COAD: Colon adenocarcinoma
DLBC: Lymphoid neoplasm diffuse large B cell lymphoma
ESCA: Esophageal carcinoma
GBM: Glioblastoma multiforme
HNSC: Head and neck squamous cell carcinoma
KICH: Kidney chromophobe
KIRC: Kidney renal clear cell carcinoma
KIRP: Kidney renal papillary cell carcinoma
LAML: Acute myeloid leukemia
LGG: Brain Lower Grade Glioma
LIHC: Liver hepatocellular carcinoma
LUAD: Lung adenocarcinoma
LUSC: Lung squamous cell carcinoma
MESO: Mesothelioma
OV: Ovarian serous cystadenocarcinoma
PAAD: Pancreatic adenocarcinoma
PCPG: Pheochromocytoma and paraganglioma
PRAD: Prostate adenocarcinoma;
READ: Rectum adenocarcinoma
SARC: Sarcoma
SKCM: Skin cutaneous melanoma
STAD: Stomach adenocarcinoma
TGCT: Testicular germ cell tumors
THCA: Thyroid carcinoma
THYM: Thymoma
UCEC: Uterine corpus endometrial carcinoma
UCS: Uterine carcinosarcoma
UVM: Uveal melanoma
