# Supplementary figures and images for: A comprehensive prognostic and immunological analysis of ephrin family genes in hepatocellular carcinoma
Source: Front Mol Biosci. 2022 Aug 16;9:943384. doi: 10.3389/fmolb.2022.943384 (PMC9424725; doi:10.3389/fmolb.2022.943384)

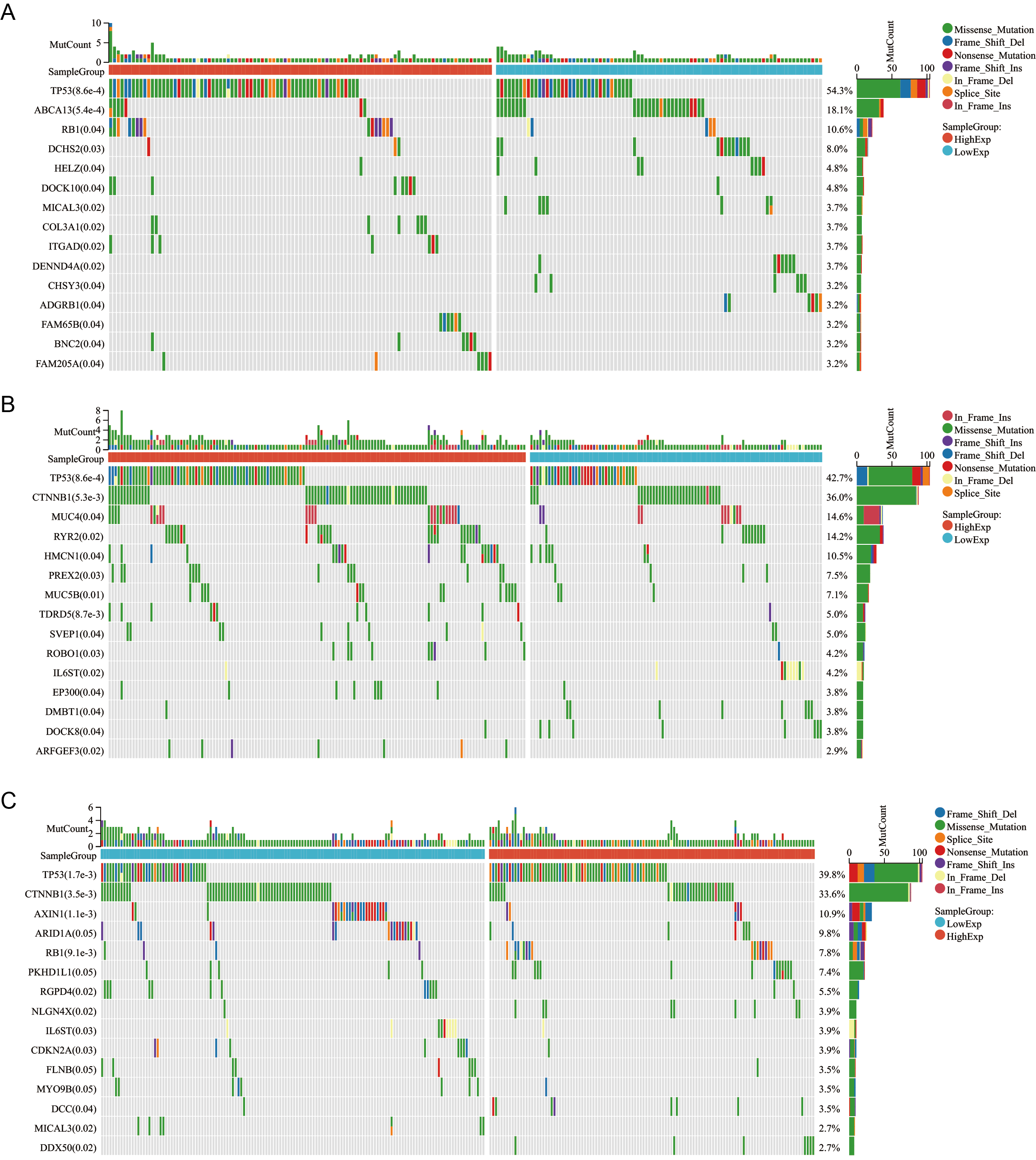

Supplement: Supplementary file 1 [file Image6.TIF]

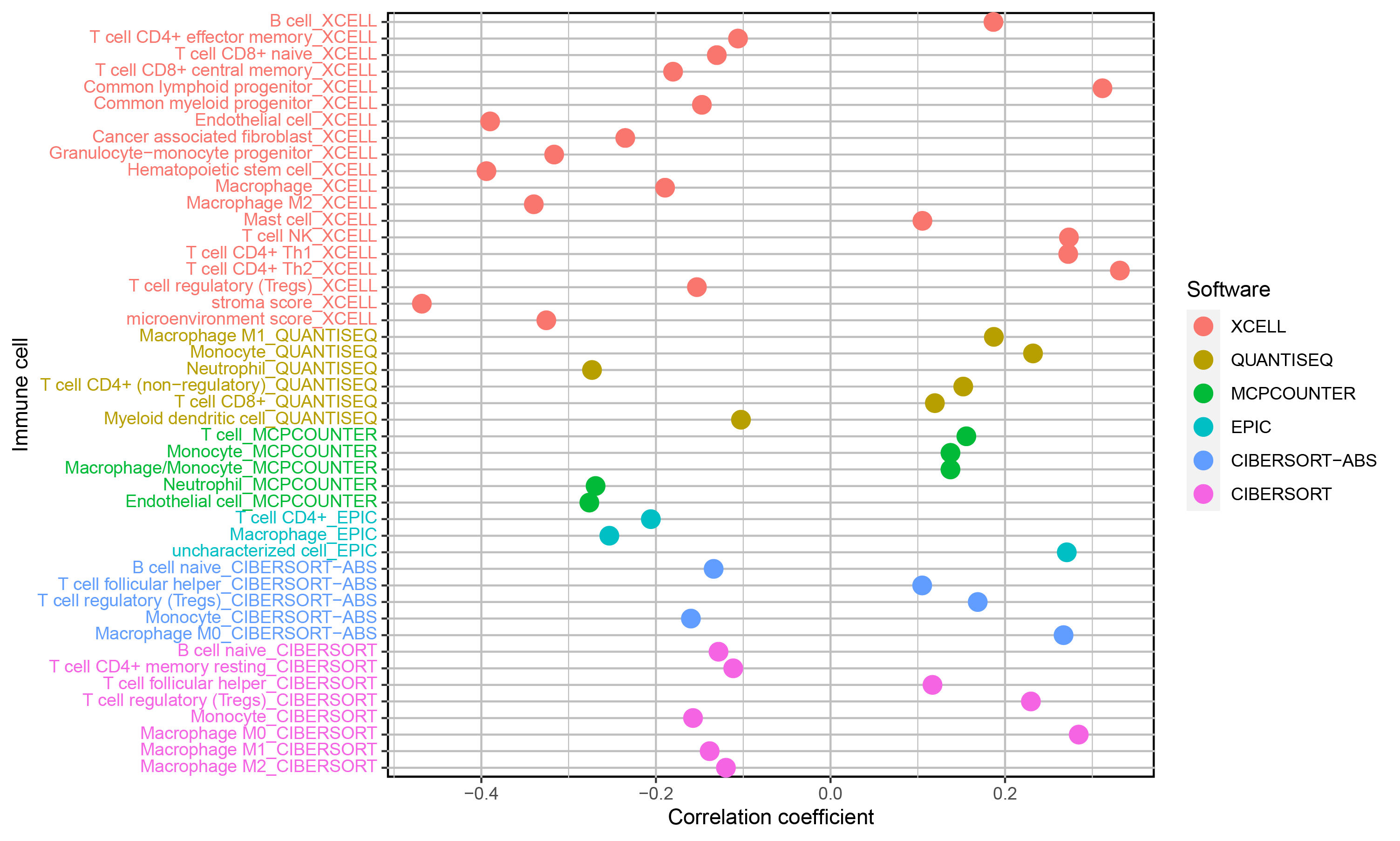

Supplement: Supplementary file 3 [file Image3.TIF]

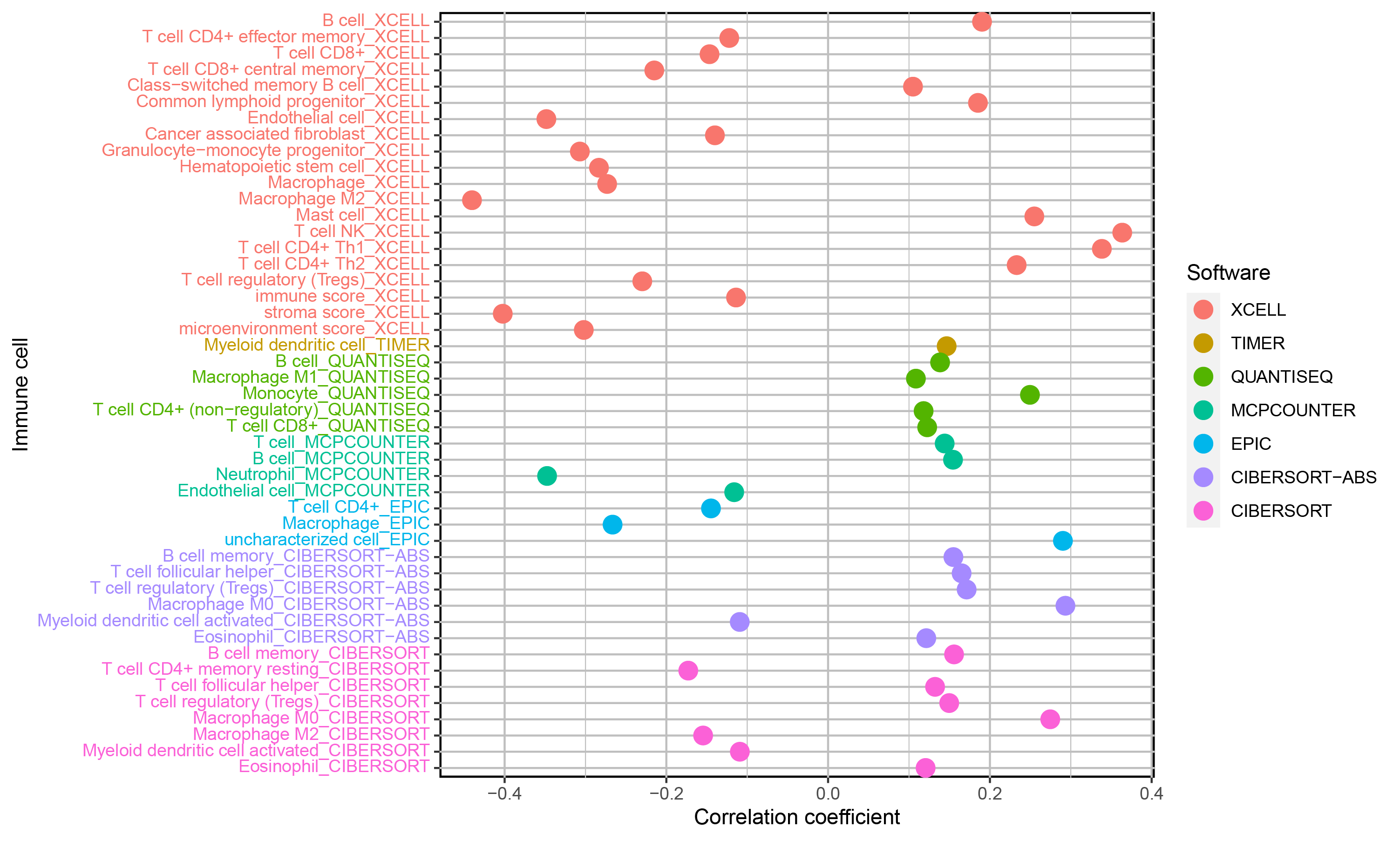

Supplement: Supplementary file 4 [file Image4.TIF]

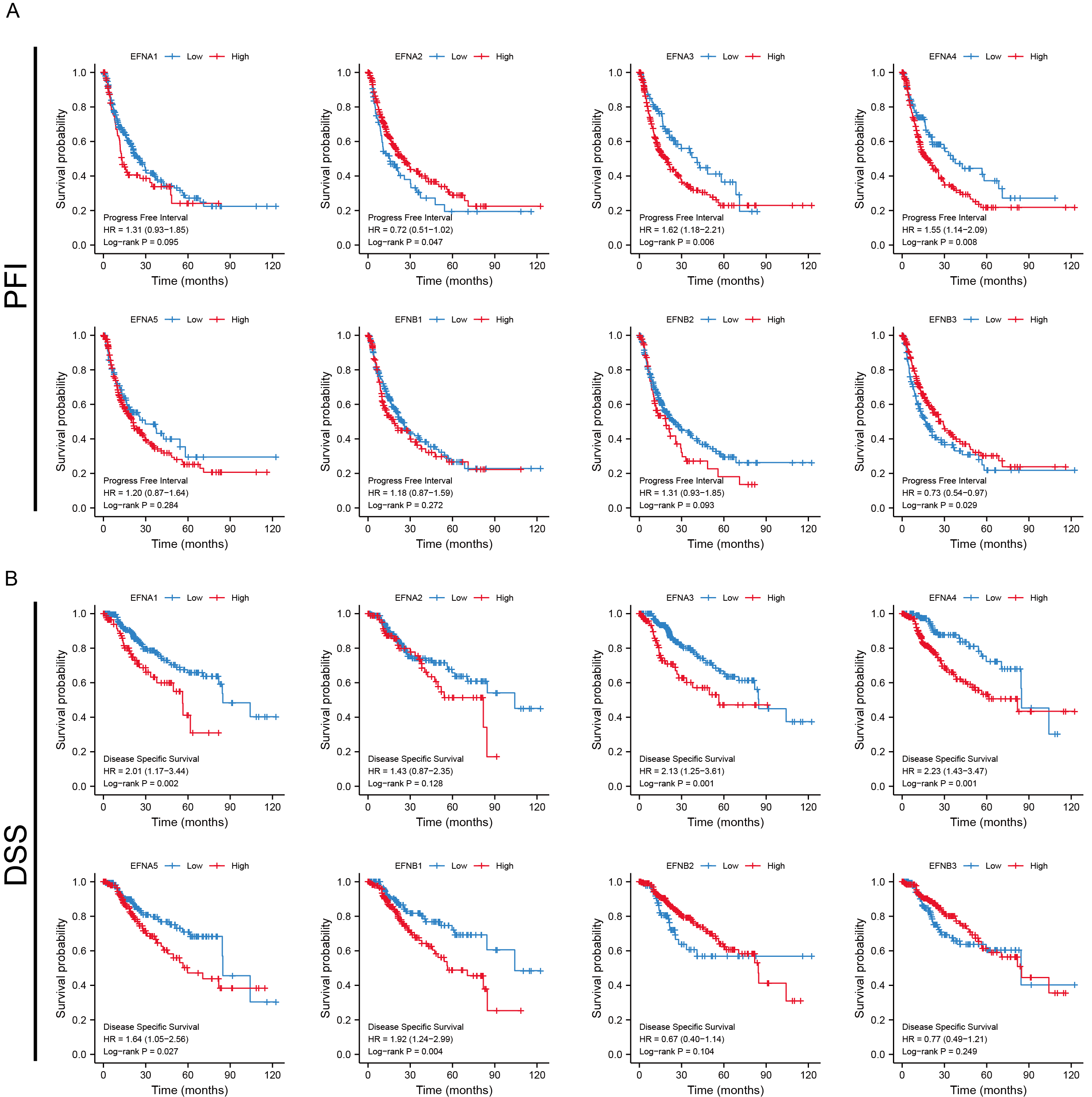

Supplement: Supplementary file 5 [file Image2.TIF]

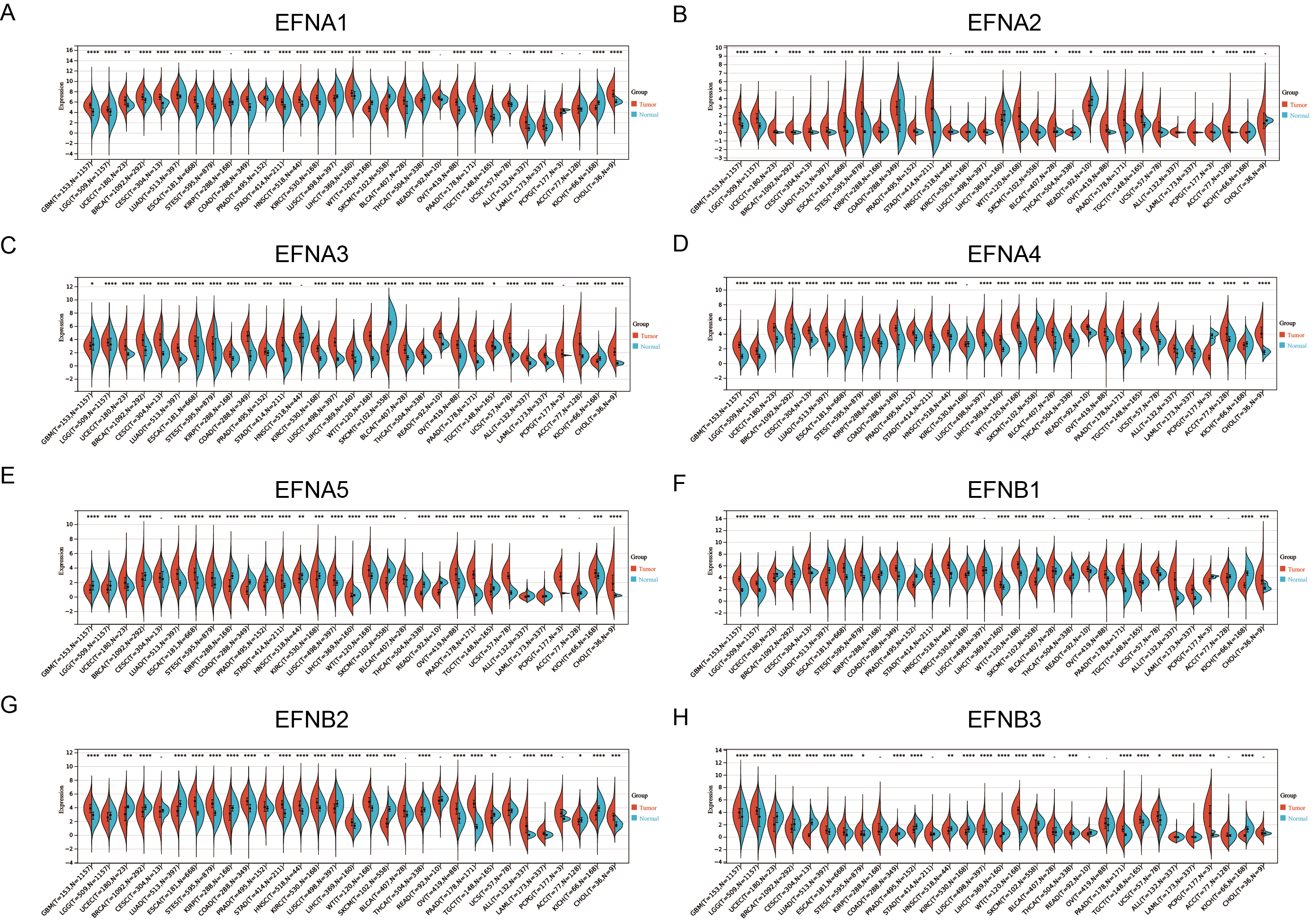

Supplement: Supplementary file 6 [file Image1.TIF]

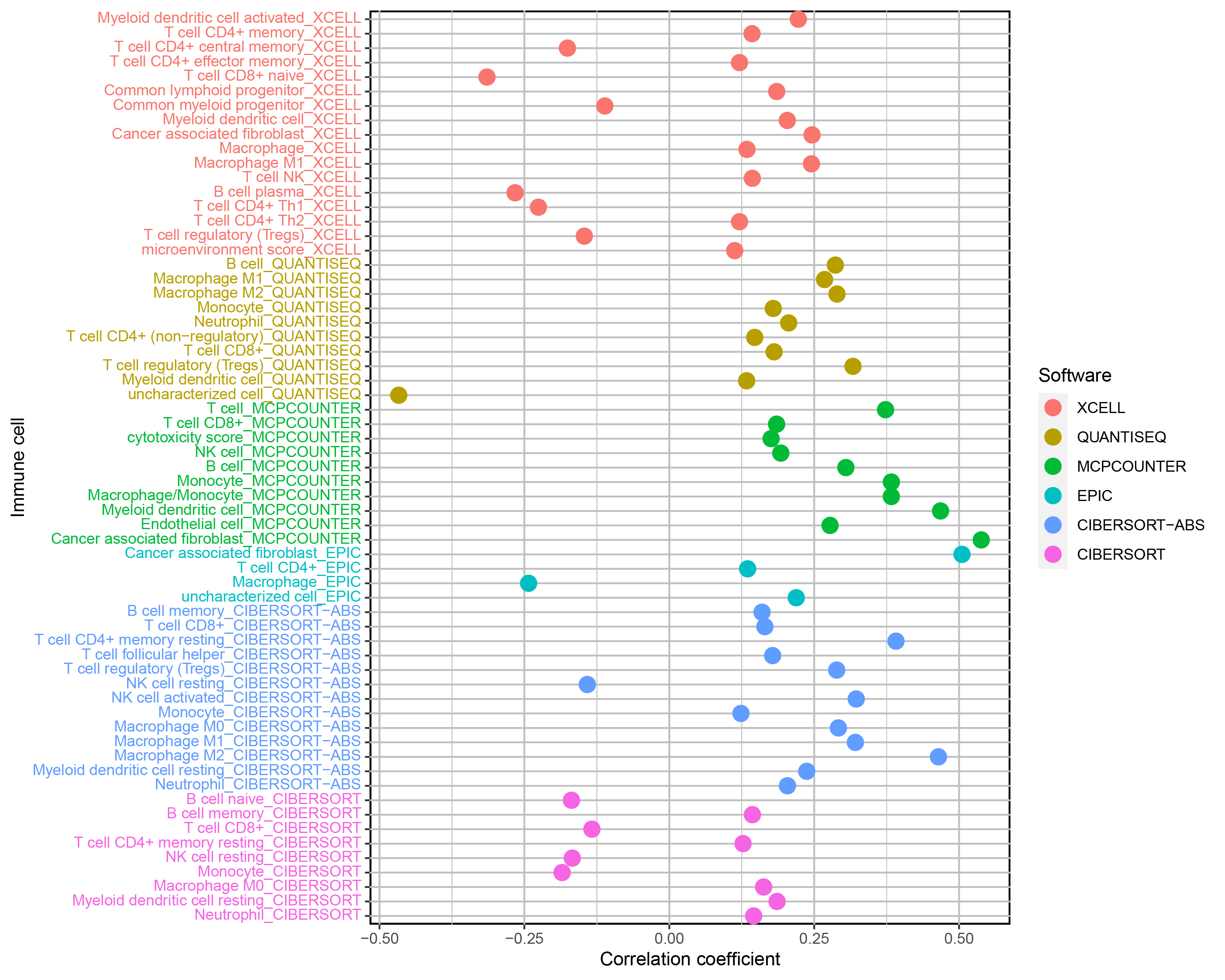

Supplement: Supplementary file 9 [file Image5.TIF]
