# Supplementary material for: A comprehensive prognostic and immunological analysis of ephrin family genes in hepatocellular carcinoma
Source: Front Mol Biosci. 2022 Aug 16;9:943384. doi: 10.3389/fmolb.2022.943384 (PMC9424725; doi:10.3389/fmolb.2022.943384)
Supplement: Supplementary file 2 [file Table1.DOCX]

**Table S1.** The correlation between EFNA3 expression and tumor immunity based on

different immune algorithms

| **Algorithms** | **Immune** | **coefficients** | ***p*-value** |
| --- | --- | --- | --- |
| CIBERSORT | B cell naïve | -0.128 | 0.013 |
| CIBERSORT | T cell CD4+ memory resting | -0.111 | 0.032 |
| CIBERSORT | T cell follicular helper | 0.117 | 0.024 |
| CIBERSORT | T cell regulatory (Tregs) | 0.229 | 7.93E-06 |
| CIBERSORT | Monocyte | -0.157 | 0.002 |
| CIBERSORT | Macrophage M0 | 0.284 | 2.48E-08 |
| CIBERSORT | Macrophage M1 | -0.138 | 0.007 |
| CIBERSORT | Macrophage M2 | -0.12 | 0.021 |
| CIBRSORT-ABS | B cell naïve | -0.134 | 0.009 |
| CIBRSORT-ABS | T cell follicular helper | 0.105 | 0.043 |
| CIBRSORT-ABS | T cell regulatory (Tregs) | 0.169 | 0.001 |
| CIBRSORT-ABS | Monocyte | -0.16 | 0.002 |
| CIBRSORT-ABS | Macrophage M0 | 0.267 | 1.78E-07 |
| QUANTISEQ | Macrophage M1 | 0.187 | 2.0E-04 |
| QUANTISEQ | Monocyte | 0.232 | 6.32E-06 |
| QUANTISEQ | Neutrophil | -0.273 | 8.94E-08 |
| QUANTISEQ | T cell CD4+ (non-regulatory) | 0.152 | 0.003 |
| QUANTISEQ | T cell CD8+ | 0.12 | 0.021 |
| QUANTISEQ | Myeloid dendritic cell | -0.102 | 0.048 |
| MCPCOUNTER | T cell | 0.156 | 0.003 |
| MCPCOUNTER | Monocyte | 0.137 | 0.008 |
| MCPCOUNTER | Macrophage/Monocyte | 0.137 | 0.008 |
| MCPCOUNTER | Neutrophil | -0.269 | 1.58E-07 |
| MCPCOUNTER | Endothelial cell | -0.276 | 7.18E-08 |
| XCELL | B cell | 0.187 | 0.0003 |
| XCELL | T cell CD4+ effector memory | -0.106 | 0.041 |
| XCELL | T cell CD8+ naïve | -0.13 | 0.012 |
| XCELL | T cell CD8+ central memory | -0.18 | 4.0E-04 |
| XCELL | Common lymphoid progenitor | 0.311 | 8.53E-10 |
| XCELL | Common myeloid progenitor | -0.147 | 0.004 |
| XCELL | Endothelial cell | -0.39 | 6.36E-15 |
| XCELL | Cancer associated fibroblast | -0.235 | 4.70E-06 |
| XCELL | Granulocyte-monocyte progenitor | -0.317 | 4.41E-10 |
| XCELL | Hematopoietic stem cell | -0.394 | 3.06E-15 |
| XCELL | Macrophage | -0.19 | 2.41E-04 |
| XCELL | Macrophage M2 | -0.34 | 1.74E-11 |
| XCELL | Mast cell | 0.105 | 0.043 |
| XCELL | T cell NK | 0.273 | 9.02E-08 |
| XCELL | T cell CD4+ Th1 | 0.272 | 9.89E-08 |
| XCELL | T cell CD4+ Th2 | 0.332 | 5.76E-11 |
| XCELL | T cell regulatory (Tregs) | -0.153 | 0.003 |
| XCELL | stroma score | -0.468 | <2.2E-16 |
| XCELL | microenvironment score | -0.326 | 1.68E-10 |
| EPIC | T cell CD4+ | -0.206 | 6.74E-05 |
| EPIC | Macrophage | -0.253 | 8.31E-07 |
| EPIC | uncharacterized cell | 0.271 | 1.33E-07 |
