# Supplementary material for: A comprehensive prognostic and immunological analysis of ephrin family genes in hepatocellular carcinoma
Source: Front Mol Biosci. 2022 Aug 16;9:943384. doi: 10.3389/fmolb.2022.943384 (PMC9424725; doi:10.3389/fmolb.2022.943384)
Supplement: Supplementary file 7 [file Table2.DOCX]

**Table S2.** The correlation between EFNA4 expression and tumor immunity based on

different immune algorithms

| **Algorithms** | **Immune** | **coefficients** | ***p*-value** |
| --- | --- | --- | --- |
| CIBERSORT | B cell memory | 0.156 | 0.003 |
| CIBERSORT | T cell CD4+ memory resting | -0.173 | 8.24E-04 |
| CIBERSORT | T cell follicular helper | 0.132 | 0.011 |
| CIBERSORT | T cell regulatory (Tregs) | 0.150 | 0.004 |
| CIBERSORT | Macrophage M0 | 0.275 | 7.63E-08 |
| CIBERSORT | Macrophage M2 | -0.155 | 0.003 |
| CIBERSORT | Myeloid dendritic cell activated | -0.109 | 0.036 |
| CIBERSORT | Eosinophil | 0.121 | 0.02 |
| CIBERSORT-ABS | B cell memory | 0.155 | 0.003 |
| CIBERSORT-ABS | T cell follicular helper | 0.165 | 0.001 |
| CIBERSORT-ABS | T cell regulatory (Tregs) | 0.171 | 9.26E-04 |
| CIBERSORT-ABS | Macrophage M0 | 0.293 | 8.43E-09 |
| CIBERSORT-ABS | Myeloid dendritic cell activated | -0.109 | 0.035 |
| CIBERSORT-ABS | Eosinophil | 0.121 | 0.019 |
| QUANTISEQ | B cell | 0.138 | 0.008 |
| QUANTISEQ | Macrophage M1 | 0.109 | 0.037 |
| QUANTISEQ | Monocyte | 0.250 | 1.13E-06 |
| QUANTISEQ | T cell CD4+ (non-regulatory | 0.118 | 0.023 |
| QUANTISEQ | T cell CD8+ | 0.122 | 0.018 |
| MCPCOUNTER | T cell | 0.144 | 0.005 |
| MCPCOUNTER | B cell | 0.154 | 0.003 |
| MCPCOUNTER | Neutrophil | -0.347 | 7.79E-12 |
| MCPCOUNTER | Endothelial cell | -0.116 | 0.025 |
| XCELL | B cell_ | 0.190 | 2.2E-04 |
| XCELL | T cell CD4+ effector memory | -0.122 | 0.019 |
| XCELL | T cell CD8+ | -0.147 | 0.005 |
| XCELL | T cell CD8+ central memory | -0.215 | 2.98E-05 |
| XCELL | Class-switched memory B cell | 0.105 | 0.043 |
| XCELL | Common lymphoid progenitor | 0.185 | 3.34E-04 |
| XCELL | Endothelial cell_ | -0.348 | 5.04E-12 |
| XCELL | Cancer associated fibroblast | -0.140 | 0.007 |
| XCELL | Granulocyte-monocyte progenitor | -0.307 | 1.56E-09 |
| XCELL | Hematopoietic stem cell | -0.283 | 2.81E-08 |
| XCELL | Macrophage | -0.273 | 8.93E-08 |
| XCELL | Macrophage M2 | -0.440 | 5.01E-19 |
| XCELL | Mast cell | 0.255 | 6.48E-07 |
| XCELL | T cell NK | 0.364 | 4.84E-13 |
| XCELL | T cell CD4+ Th1 | 0.338 | 2.15E-11 |
| XCELL | T cell CD4+ Th2 | 0.233 | 5.69E-06 |
| XCELL | T cell regulatory (Tregs) | -0.230 | 7.80E-06 |
| XCELL | immune score | -0.114 | 0.028383 |
| XCELL | stroma score | -0.402 | 0 |
| XCELL | microenvironment score | -0.302 | 3.47E-09 |
| EPIC | T cell CD4+ | -0.145 | 0.005 |
| EPIC | Macrophage | -0.266 | 2.10E-07 |
| EPIC | uncharacterized cell | 0.290 | 1.43E-08 |
