# Supplementary material for: A comprehensive prognostic and immunological analysis of ephrin family genes in hepatocellular carcinoma
Source: Front Mol Biosci. 2022 Aug 16;9:943384. doi: 10.3389/fmolb.2022.943384 (PMC9424725; doi:10.3389/fmolb.2022.943384)
Supplement: Supplementary file 8 [file Table3.DOCX]

**Table S3.** The correlation between EFNB1 expression and tumor immunity based on

different immune algorithms

| **Algorithms** | **Immune** | **coefficients** | ***p*-value** |
| --- | --- | --- | --- |
| CIBERSORT | B cell naive | -0.169 | 0.001 |
| CIBERSORT | B cell memory | 0.143 | 0.006 |
| CIBERSORT | T cell CD8+ | -0.134 | 0.01 |
| CIBERSORT | T cell CD4+ memory resting | 0.127 | 0.014 |
| CIBERSORT | NK cell resting | -0.167 | 0.001 |
| CIBERSORT | Monocyte | -0.185 | 3.40E-04 |
| CIBERSORT | Macrophage M0 | 0.163 | 0.002 |
| CIBERSORT | Myeloid dendritic cell resting_ | 0.186 | 3.16E-04 |
| CIBERSORT | Neutrophil_ | 0.145 | 0.005 |
| CIBERSORT-ABS | B cell memory_ | 0.160 | 0.002 |
| CIBERSORT-ABS | T cell CD8+ | 0.165 | 0.001 |
| CIBERSORT-ABS | T cell CD4+ memory resting | 0.391 | 5.23E-15 |
| CIBERSORT-ABS | T cell follicular helper | 0.178 | 5.6E-04 |
| CIBERSORT-ABS | T cell regulatory (Tregs) | 0.289 | 1.46E-08 |
| CIBERSORT-ABS | NK cell resting | -0.141 | 0.006 |
| CIBERSORT-ABS | NK cell activated | 0.323 | 1.99E-10 |
| CIBERSORT-ABS | Monocyte | 0.124 | 0.017 |
| CIBERSORT-ABS | Macrophage M0 | 0.292 | 1.04E-08 |
| CIBERSORT-ABS | Macrophage M1 | 0.321 | 2.45E-10 |
| CIBERSORT-ABS | Macrophage M2 | 0.464 | <2.2E-16 |
| CIBERSORT-ABS | Myeloid dendritic cell resting | 0.237 | 3.88E-06 |
| CIBERSORT-ABS | Neutrophil | 0.204 | 7.66E-05 |
| QUANTISEQ | B cell | 0.286 | 1.94E-08 |
| QUANTISEQ | Macrophage M1 | 0.268 | 1.63E-07 |
| QUANTISEQ | Macrophage M2 | 0.289 | 1.38E-08 |
| QUANTISEQ | Monocyte | 0.179 | 5.2E-04 |
| QUANTISEQ | Neutrophil | 0.206 | 6.41E-05 |
| QUANTISEQ | T cell CD4+ (non-regulatory) | 0.147 | 0.004 |
| QUANTISEQ | T cell CD8+ | 0.181 | 4.67E-04 |
| QUANTISEQ | T cell regulatory (Tregs) | 0.317 | 4.23E-10 |
| QUANTISEQ | Myeloid dendritic cell | 0.133 | 0.01 |
| QUANTISEQ | uncharacterized cell | -0.467 | <2.2E-16 |
| MCPCOUNTER | T cell | 0.373 | 1.40E-13 |
| MCPCOUNTER | T cell CD8+ | 0.185 | 3.36E-04 |
| MCPCOUNTER | cytotoxicity score | 0.176 | 6.92E-04 |
| MCPCOUNTER | NK cell | 0.192 | 1.99E-04 |
| MCPCOUNTER | B cell | 0.305 | 2.52E-09 |
| MCPCOUNTER | Monocyte | 0.383 | 2.34E-14 |
| MCPCOUNTER | Macrophage/Monocyte | 0.383 | 2.34E-14 |
| MCPCOUNTER | Myeloid dendritic cell | 0.468 | <2.2E-16 |
| MCPCOUNTER | Endothelial cell | 0.277 | 6.41E-08 |
| MCPCOUNTER | Cancer associated fibroblast | 0.538 | <2.2E-16 |
| XCELL | Myeloid dendritic cell activatedL | 0.223 | 1.50E-05 |
| XCELL | T cell CD4+ memory | 0.143 | 0.006 |
| XCELL | T cell CD4+ central memory | -0.176 | 6.7E-04 |
| XCELL | T cell CD4+ effector memory | 0.121 | 0.02 |
| XCELL | T cell CD8+ naive | -0.315 | 5.73E-10 |
| XCELL | Common lymphoid progenitor | 0.185 | 3.3E-04 |
| XCELL | Common myeloid progenitor | -0.111 | 0.032 |
| XCELL | Myeloid dendritic cell | 0.204 | 7.86E-05 |
| XCELL | Cancer associated fibroblast | 0.246 | 1.57E-06 |
| XCELL | Macrophage | 0.134 | 0.01 |
| XCELL | Macrophage M1 | 0.245 | 1.74E-06 |
| XCELL | T cell NK | 0.143 | 0.006 |
| XCELL | B cell plasma | -0.266 | 1.98E-07 |
| XCELL | T cell CD4+ Th1 | -0.226 | 1.12E-05 |
| XCELL | T cell CD4+ Th2 | 0.121 | 0.02 |
| XCELL | T cell regulatory (Tregs) | -0.147 | 0.005 |
| XCELL | microenvironment score | 0.113 | 0.03 |
| EPIC | Cancer associated fibroblast | 0.505 | <2.2E-16 |
| EPIC | T cell CD4+ | 0.135 | 0.009 |
| EPIC | Macrophage | -0.243 | 2.40E-06 |
| EPIC | uncharacterized cell | 0.219 | 2.19E-05 |
